# Metabolic Inhibitors of O‐GlcNAc Transferase That Act In Vivo Implicate Decreased O‐GlcNAc Levels in Leptin‐Mediated Nutrient Sensing

**DOI:** 10.1002/anie.201803254

**Published:** 2018-05-24

**Authors:** Tai‐Wei Liu, Wesley F. Zandberg, Tracey M. Gloster, Lehua Deng, Kelsey D. Murray, Xiaoyang Shan, David J. Vocadlo

**Affiliations:** ^1^ Departments of Chemistry & Molecular Biology and Biochemistry Simon Fraser University 8888 University Dr. Burnaby BC Canada; ^2^ Current address: Department of Chemistry University of British Columbia 1177 Research Road Kelowna BC Canada; ^3^ Current address: School of Biology Biomedical Sciences Research Complex University of St Andrews North Haugh St Andrews Fife UK

**Keywords:** glycoproteins, inhibitors, leptin, nucleotide sugars, thiosugars

## Abstract

O‐Linked glycosylation of serine and threonine residues of nucleocytoplasmic proteins with N‐acetylglucosamine (O‐GlcNAc) residues is catalyzed by O‐GlcNAc transferase (OGT). O‐GlcNAc is conserved within mammals and is implicated in a wide range of physiological processes. Herein, we describe metabolic precursor inhibitors of OGT suitable for use both in cells and in vivo in mice. These 5‐thiosugar analogues of N‐acetylglucosamine are assimilated through a convergent metabolic pathway, most likely involving N‐acetylglucosamine‐6‐phosphate de‐N‐acetylase (NAGA), to generate a common OGT inhibitor within cells. We show that of these inhibitors, 2‐deoxy‐2‐N‐hexanamide‐5‐thio‐d‐glucopyranoside (5SGlcNHex) acts in vivo to induce dose‐ and time‐dependent decreases in O‐GlcNAc levels in various tissues. Decreased O‐GlcNAc correlates, both in vitro within adipocytes and in vivo within mice, with lower levels of the transcription factor Sp1 and the satiety‐inducing hormone leptin, thus revealing a link between decreased O‐GlcNAc levels and nutrient sensing in peripheral tissues of mammals.

The dynamic modification of nucleocytoplasmic proteins with β‐O‐linked *N*‐acetylglucosamine (O‐GlcNAc) is present in all multicellular eukaryotes.^1^ The attachment of GlcNAc (**1**, Figure 1) to hydroxy groups of serine or threonine residues of hundreds of target proteins is catalyzed by the glycosyltransferase O‐GlcNAc transferase (OGT), which uses uridine diphospho‐*N*‐acetylglucosamine (UDP‐GlcNAc, **2**) as a donor sugar substrate. The glycosidase O‐GlcNAcase (OGA) cleaves O‐GlcNAc off from proteins.

**Figure 1:**
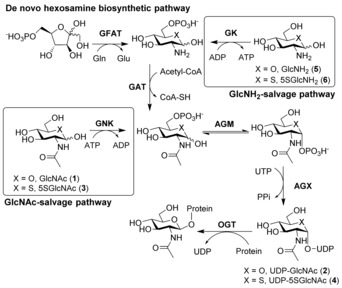
The hexosamine biosynthetic pathway (HBP) and the GlcNAc salvage pathway yield UDP‐GlcNAc (**2**). Glutamine fructose amidotransferase (GFAT) catalyzes the conversion of glucosamine (**5**) into glucosamine‐6‐phosphate (GlcNH_2_‐6PO_4_; **5**‐6PO_4_). Acetylation of **5**‐6PO_4_ by glucosamine acetyltransferase (GAT) yields GlcNAc‐6PO_4_, an intermediate also produced by GlcNAc kinase (GNK) in the GlcNAc salvage pathway. The sequential action of phosphoglucosamine mutase (AGM) and GlcNAc pyrophosphorylase (AGX) on GlcNAc‐6PO_4_ leads to the formation of UDP‐GlcNAc (**2**). 5SGlcNAc (**3**) can be similarly salvaged and converted into UDP‐5SGlcNAc (**4**).

O‐GlcNAc is implicated in various diseases including cancer, neurodegeneration, cardiovascular disease, and obesity.^2^ Notably, O‐GlcNAc levels respond to nutrient availability both within cells and in vivo in animal models.1b Transgenic mice overexpressing OGT in fat or muscle tissue exhibit elevated serum leptin and insulin levels in addition to insulin resistance.^3^ Furthermore, deletion of the gene encoding OGT from neurons of the paraventricular nucleus (PVN) within the hypothalamus of mice results in uncontrolled eating.2b

Notably, OGA inhibitors that are active in vivo have helped gain insight into the roles of increased O‐GlcNAc levels in mammals. Strikingly, OGA inhibitors sometimes yield different results from those made using genetic approaches to increase O‐GlcNAc levels,^4^ perhaps because OGT and OGA also have non‐catalytic roles. Unfortunately, similar studies regarding the influence of decreased O‐GlcNAc on mammalian physiology are lacking because there are no OGT inhibitors suitable for use in vivo. Given the emerging roles of OGT in nutrient sensing and other processes, inhibitors of OGT that can be used as research tools in vivo are of high interest.^5^

One approach to decreasing O‐GlcNAc levels using small molecules has been to use broad‐spectrum amidotransferase inhibitors such as 6‐diazo‐5‐oxo‐l‐norleucine (DON), which promiscuously blocks all amidotransferases that biosynthesize many cellular metabolites including UDP‐GlcNAc. High‐throughput screening has been pursued to deliver hits that can be improved upon.5b,5d These leads, however, show modest cellular activity and limited solubility.5b They also exhibit off‐target cellular toxicity.5b Another approach5c has been to generate a GlcNAc analogue, 2‐acetamido‐2‐deoxy‐5‐thio‐α‐d‐glucopyranose (5SGlcNAc, **3**), which in its per‐O‐acetylated form (Ac_4_5SGlcNAc, **3**‐OAc) can diffuse across the plasma membrane. Within cells, **3**‐OAc is de‐O‐acetylated and assimilated via the GlcNAc salvage pathway (Figure 1) to generate UDP‐5SGlcNAc (**4**), which is a competitive OGT inhibitor (*K*_i_=8 μm). **3**‐OAc lowers cellular O‐GlcNAc levels (EC_50_=0.8–5 μm). This metabolic OGT inhibitor is not toxic but suffers from poor solubility in aqueous solution. Indeed, while often used in cell studies,^6^ solubilizing **3**‐OAc requires high concentrations of DMSO, making it incompatible for dosing of mammals. Accordingly, to explore the roles of decreased O‐GlcNAc levels in organismal physiology, compounds that act in vivo are of high interest.

To generate a tool compound for inhibiting OGT in vivo, we synthesized a panel of water‐soluble analogues of 5SGlcNAc (**3**) possessing various *N*‐acyl substituents (**7**–**17**; Figure 2 a, b and Scheme S1 in the Supporting Information). We reasoned that these hydrophobic *N*‐acyl groups would confer a balance between hydrophilicity and lipophilicity, making them water‐soluble yet able to diffuse into cells. Furthermore, although the substrate specificity of *N*‐acetylglucosamine‐6‐phosphate de‐N‐acetylase (NAGA) is not known,^7^ we speculated that compounds **7**–**17**, once phosphorylated, might be substrates for this recently identified enzyme (Figure 2 b). Action of NAGA on phosphorylated **7**–**17** would lead to formation of a common intermediate, 5‐thioglucosamine‐6‐phosphate (**5**‐6PO_4_), which can be assimilated by the hexosamine biosynthetic pathway (HBP). However, direct entry of **7**–**17** into the GlcNAc salvage pathway cannot be ruled out as the specificity of NAGA is unknown, and bulkier *N*‐glycolyl (GlcNGc)‐ and *N*‐azidoacetyl (GlcNAz)‐containing analogues enter the HBP.^8^

**Figure 2:**
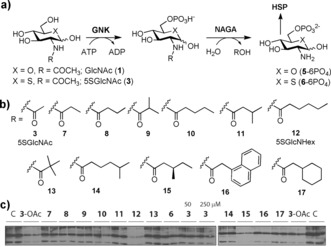
Synthetic analogues of 5SGlcNAc (**3**) are metabolized within cells, leading to decreased O‐GlcNAc levels in cells. a) Possible metabolism of 5SGlcNR analogues (**7**–**17**) by NAGA prior to their entry into the HBP. b) Synthetic analogues (**7**–**17**) bearing various *N*‐acyl R groups. c) Effects of analogues on the O‐GlcNAc levels of HEK293 cells. C=vehicle (PBS alone).

To measure the potential of **7**–**17** and their per‐O‐acetylated congeners to block OGT activity in cells, we treated cells with these compounds. Analysis of O‐GlcNAc levels of cell lysates showed that only some deprotected analogues were effective in reducing O‐GlcNAc levels (Figure 2 c and Figure S1). Of these, the promising 2‐hexanamide derivative (5SGlcNHex, **12**) showed dose‐dependent decreases in O‐GlcNAc comparable to those achieved with Ac_4_5SGlcNAc (**3**‐OAc; Figure S2).

We next tested whether these compounds were directly activated as UDP‐linked analogues or if they were metabolized by NAGA. We therefore analyzed the pool of nucleotide sugars from cells treated with compounds **7**, **8**, and **12** by capillary electrophoresis (CE; Figure 3). Electropherograms for nucleotide sugars from cells treated with **7**, **8**, and **12** revealed two new peaks with CE mobilities matching those of the chemoenzymatically prepared standards UDP‐5SGlcNAc (**4**) and its epimer UDP‐5SGalNAc (Figure 3 a). Extracts from cells treated with per‐O‐acetylated 5SGlcNH_2_ (**6**‐OAc, Figure S3) also yielded the same two unnatural nucleotide sugars in cells. These data suggest that these 5SGlcNR analogues are processed within cells to the common intermediate 2‐amino‐2‐deoxy‐5‐thioglucopyranose 6‐phosphate (**6**‐PO_4_) and then assimilated by the HBP to form UDP‐5SGlcNAc (**4**).

**Figure 3:**
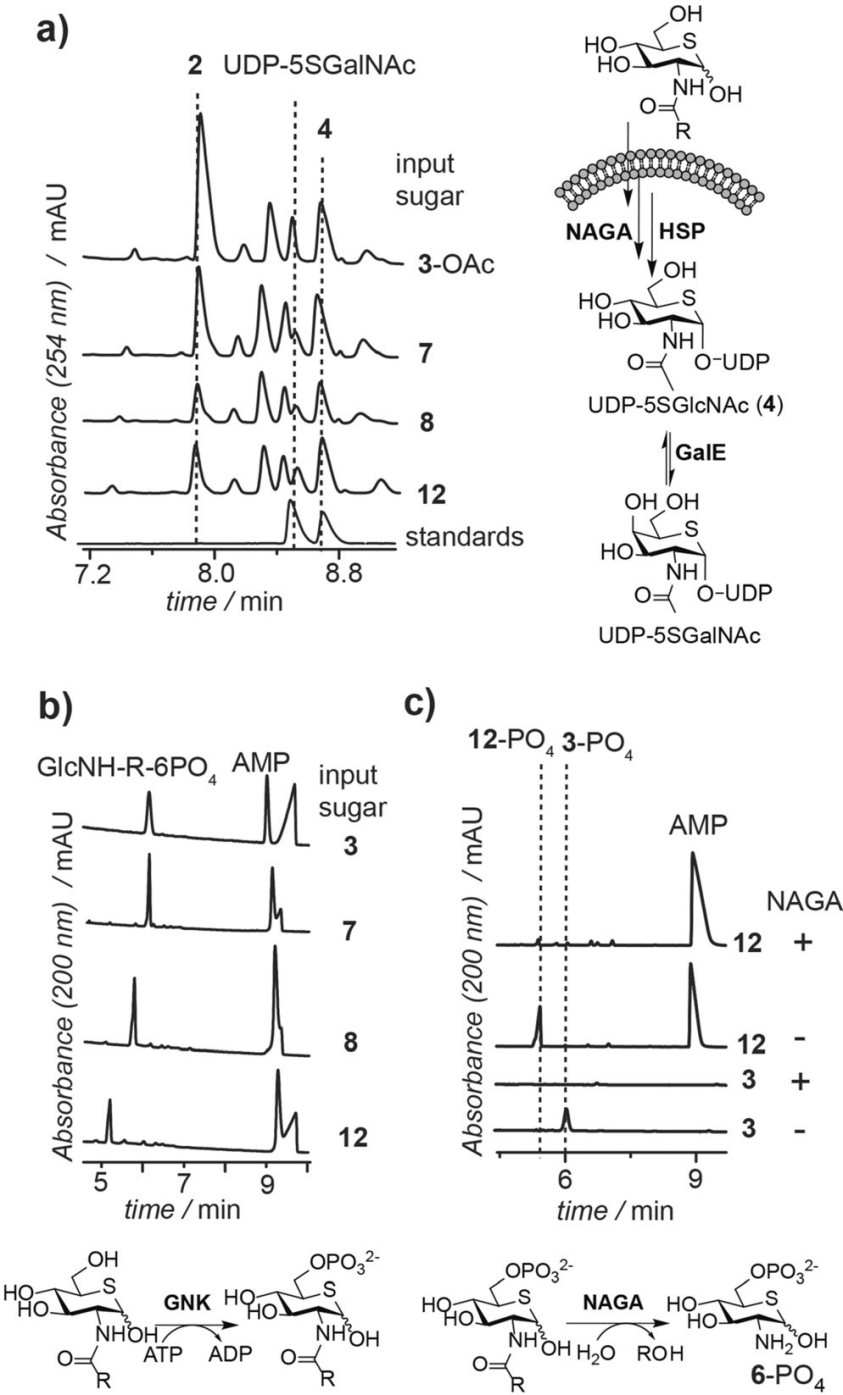
5SGlcNR analogues are sequentially processed in cells by GlcNAc kinase (GNK) and, likely, *N*‐acetylglucosamine‐6‐phosphate‐de‐N‐acetylase (NAGA) to generate 5SGlcNH_2_‐6PO_4_ (**6**‐PO_4_). a) Nucleotide sugar analysis of cells treated with selected inhibitors indicated that all of the 5SGlcNR analogues tested (**3**‐OAc, **7**, **8**, and **12**) were converted within cells into two new nucleotide sugars, indistinguishable from synthetically prepared UDP‐5SGlcNAc (**4**) and UDP‐5SGalNAc. b) 5SGlcNR analogues were phosphorylated by GNK in vitro. c) 5SGlcNR‐6‐phosphates were not substrates for AGM or AGX but were readily hydrolyzed in vitro by NAGA to produce 5SGlcNH2‐6PO_4_ (**6**‐PO_4_).

To clarify the metabolic processing of compounds **7**, **8**, and **12**, we used recombinant enzymes of the mammalian HBP (Figure 1). Compounds **7**, **8**, and **12** were all phosphorylated by GNK (Figure 3 b), yet none of the phosphorylated products were substrates for AGM except for **7**. However, the nucleotide sugar produced in vitro from **7** by the combined HBP enzymes had a different mobility than nucleotide sugar UDP‐5SGlcNAc (**4**), which we detected in cells treated with compound **7** (Figure S4). We accordingly tested whether NAGA converted the corresponding 5‐thiosugar‐6‐PO_4_ derivatives of **7**, **8**, and **12** into 5SGlcNH_2_‐6‐PO_4_ (**6**‐6PO_4_). We confirmed this scenario by digestion of **12**‐PO_4_ with recombinant human NAGA. Electropherograms obtained by CE analysis of the processing of **12**‐PO_4_ by NAGA showed that this compound was converted into 5SGlcNH_2_‐6‐PO_4_ (**6**‐PO_4_; Figure 3 c). These data suggest that the 5SGlcNR analogues **7**–**17**, including **7**, **8**, and the most potent derivative 5SGlcNHex (**12**), are phosphorylated within cells by GNK and then deacylated by NAGA, which we found to display remarkable substrate tolerance. The resulting common intermediate, 5SGlcNH_2_‐6‐PO_4_ (**6**‐PO_4_), is then assimilated via the HBP to form UDP‐5SGlcNAc (**4**), which leads to metabolic inhibition of OGT and decreased O‐GlcNAc levels in tissues.

Previous efforts to use Ac_4_5SGlcNAc (**3**‐OAc) in vivo in mammals failed because of its poor aqueous solubility. We therefore evaluated whether these new water‐soluble metabolic OGT inhibitors could be used in vivo. Accordingly, we dosed mice by intraperitoneal (IP) delivery with our most cell‐active compound **12**. A concentration‐dependent decrease in spleen O‐GlcNAc levels was observed upon treatment with **12**, with apparent effects at even 3 mg kg^−1^ (Figure S5 a). Compound **12** (300 mg kg^−1^) decreased the spleen O‐GlcNAc levels over time with maximal inhibition by 8 h, which was maintained for at least 48 h (Figure S5 b). Compound **12** reduced O‐GlcNAc levels in the kidneys, lungs, fat, pancreas, heart, spleen, and muscle tissue but not in the blood or brain (Figure S5 c). Mice injected with high doses of **12** (300 mg kg^−1^) became lethargic, which is a known consequence of low leptin levels.^9^ A second injection of 300 mg kg^−1^ on a second day caused mice to be moribund, and we discontinued treatment. We therefore dosed mice IP with a lower dose of **12** (50 mg kg^−1^, *n*=3) and found reduced O‐GlcNAc levels in various tissues 16 h after dosing but no effects on brain, liver, pancreas, and kidney (Figure 4 a, b and Figure S6 a–c). The O‐GlcNAc levels had returned to baseline levels after 16 h (Figure 4 a). Metabolic inhibitor **12** therefore acts in vivo to reversibly lower *O*‐GlcNAcylation in various tissue types.

**Figure 4:**
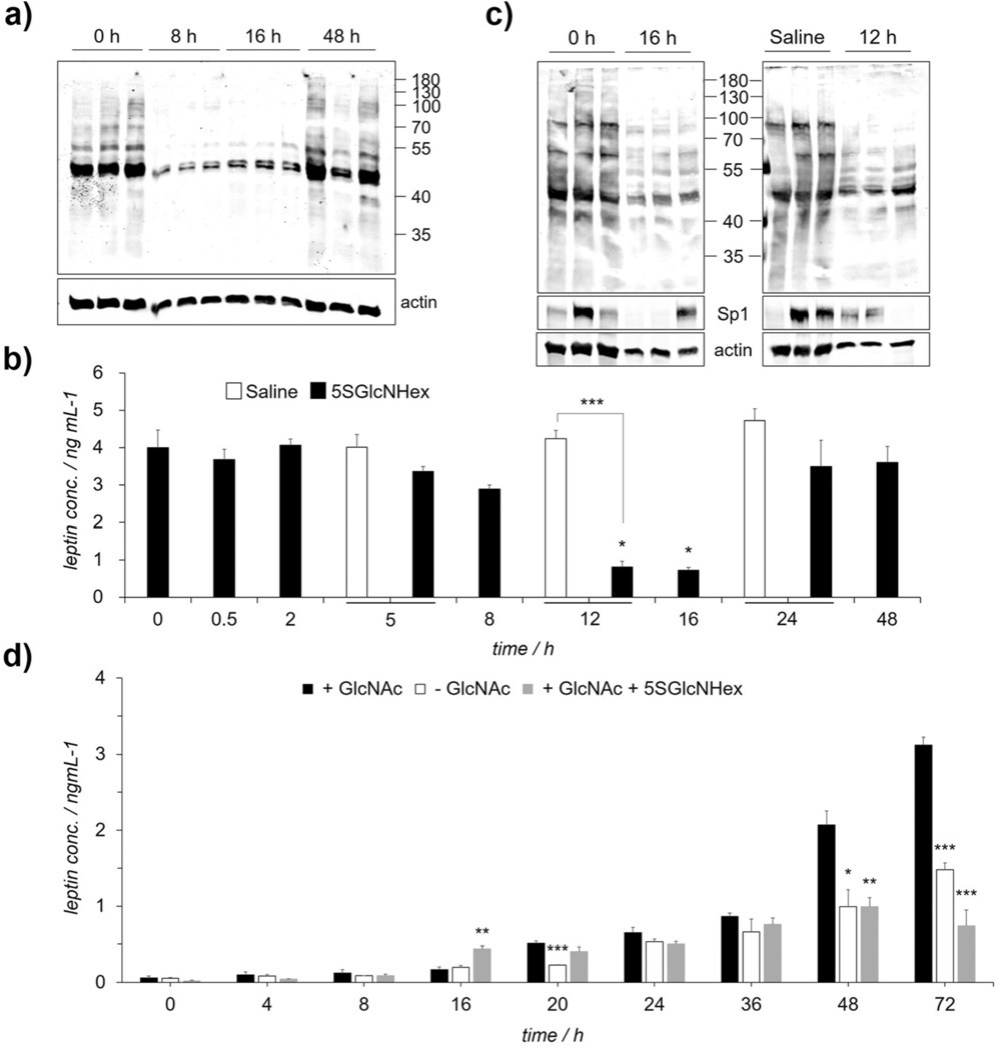
Dosing of mice with 50 mg kg^−1^ 5SGlcNHex (**12**) reduced the O‐GlcNAc levels and impaired the secretion of the hormone leptin. a) Skeletal muscle from mice dosed with 50 mg kg^−1^ of **12** showed a transient reduction in O‐GlcNAc levels by immunoblot analysis (CTD110.6). b) Leptin levels as measured by ELISA decreased in mice dosed with **12** to a minimum at 16 h. c) Dosing with **12** decreased O‐GlcNAc and Sp1 levels to a minimum at 16 h in fat pad tissue. d) Compound **12** lowered GlcNAc‐induced leptin secretion from 3T3‐L1 adipocytes. Results are given as the mean±SEM of three independent samples (*n=*3). Each independent sample was tested in duplicate; symbols denote statistical significance (*p<0.05, **p<0.01, ***p<0.001 compared to control; Student's t‐test).

Notably, others have shown that Ac_4_5SGlcNAc (**3**‐OAc) appears to be able to inhibit other glycosyltransferases in cell lines.5b We found, however, by lectin blot assessment that **12** induced no apparent changes in other forms of protein glycosylation in all tissues tested (Figure S7) even at a dose of 300 mg kg^−1^ after 48 h, which is consistent with a recent report on the use of the parent compound Ac_4_5SGlcNAc (**3**‐OAc) in cells.^10^

Curiously, we observed that mice treated with either low or high doses (50–300 mg kg^−1^) of **12** exhibited engorged stomachs that were full of rodent chow after 16 h (Figure S8). As lethargy and excessive food consumption (hyperphagia) are seen in mice deficient in leptin signaling,^11^ we measured the serum leptin concentration of animals treated with 50 mg kg^−1^ of **12** (Figure 4 b). Their leptin levels dropped transiently to a minimum at 16 h (Figure 4 b), consistent with the engorged stomachs.^9^, ^12^ The leptin levels were also lower in mice treated with a high dose of **12** (300 mg kg^−1^; Figure S9). We therefore hypothesized that OGT inhibition might impair leptin production (Figure S10).

To test this hypothesis, we used 3T3‐L1 adipocytes and found that these cells secreted less leptin when treated with either **12** (Figure 4 d) or Ac_4_5SGlcNAc (**3**‐OAc; Figure S11) without affecting glucose uptake. Notably, the transcription factors C/EBP‐α, ‐β, and Sp1 all regulate leptin production. Compound **12** lowered the levels of these known O‐GlcNAcylated proteins in adipocytes (Figures S9) and in mice (Figure S6 d). These collective data are consistent with OGT acting as a nutrient sensor in a process coupling HBP flux to leptin levels (Figure S10).

We envision that future studies to detail dosing regimens with **12** will be important to explore the effects of decreased O‐GlcNAc levels on various physiological processes. Experiments will also be needed to detail the mechanistic links between reduced O‐GlcNAc levels due to in vivo OGT inhibition, impaired leptin secretion, and apparent hyperphagia. However, our data provide the first direct correlation between decreased O‐GlcNAc levels and impaired leptin production in vivo. Our findings are in accord with studies showing that reduced O‐GlcNAc lowers levels of Sp1 in cells6f, 13 and that OGT plays a pivitol role in fat tissues.^14^ Notably, these data also suggest that regulation of leptin by O‐GlcNAc is bidirectional in vivo as overexpression of OGT,^3^ knockout of OGA,^15^ and metabolic upregulation of UDP GlcNAc levels^16^ all increase leptin expression.

In summary, we have described convenient new compounds to inhibit OGT in cells and in vivo. Strikingly, inhibition of OGT with 5SGlcNHex (**12**) provides support for the hypothesis that reduced O‐GlcNAc levels signal impaired nutrient supply in mammals. We expect that these observations will stimulate activity in the development of additional metabolic OGT inhibitors. Moreover, we envision that this chemical strategy of metabolic OGT inhibition will serve as a valuable complement to using genetic methods in various rodent models to accelerate studies to understand the in vivo roles of O‐GlcNAc in mammals.

## Experimental Section

Please see the Supporting Information for full experimental procedures. All experiments carried out on animals were approved by the University Animal Care Committee of SFU.

## Conflict of interest

These compounds are the subject matter of a provisional patent application on inhibitors of O‐GlcNAc transferase. D.J.V., L.D., and T.M.G. are inventors on this patent application.

## Supporting information

As a service to our authors and readers, this journal provides supporting information supplied by the authors. Such materials are peer reviewed and may be re‐organized for online delivery, but are not copy‐edited or typeset. Technical support issues arising from supporting information (other than missing files) should be addressed to the authors.

Supplementary
