# Supplementary material for: Metabolic Inhibitors of O‐GlcNAc Transferase That Act In Vivo Implicate Decreased O‐GlcNAc Levels in Leptin‐Mediated Nutrient Sensing
Source: Angew Chem Int Ed Engl. 2018 May 24;57(26):7644–8. doi: 10.1002/anie.201803254 (PMC6055616; doi:10.1002/anie.201803254)
Supplement: Supplementary file 1 — Supplementary [file ANIE-57-7644-s001.pdf]

Angewandte Chemie
Eine Zeitschrift der Gesellschaft Deutscher Chemiker

Supporting Information

Metabolic Inhibitors of O-GlcNAc Transferase That Act In Vivo Implicate Decreased O-GlcNAc Levels in Leptin-Mediated Nutrient Sensing

*Tai-Wei Liu[+], Wesley F. Zandberg[+], Tracey M. Gloster[+], Lehua Deng, Kelsey D. Murray, Xiaoyang Shan, and David J. Vocadlo**

anie_201803254_sm_miscellaneous_information.pdf

SUPPORTING INFORMATION

Contents **Page**

Supplemental Figures S1 – S11

Figure S1. Comparison of protected and deprotected 5SGlcNR analogues (**7-17**) in HEK293 cells...……………………………………………………………………..S4

Figure S2. Time-course experiments in CHO K1 cells demonstrate that 5SGlcNHex (**12**) and peracetylated 5SGlcNAc (**3**-OAc) prevent OGT activity with similar kinetics……………………………………………………………………...………S5

Figure S3. Quantification of nucleotide sugars extracted from 5SGlcNHex (**12)** and per-O-acetylated 5SGlcNH$_2$ (**6**-OAc)-treated cells……………………………………..…….S5

Figure S4. 5SGlcNPropyl (**7)** is a substrate for the enzymes of the GlcNAc-salvage pathway in vitro but not in cells…………………………………………………………S6

Figure S5. Exposure of mice to 5SGlcNHex (**12**) for 16 h resulted in the decrease in *O*-GlcNAc levels across multiple tissues…………………………………………………S7

Figure S6. Exposure of mice to a lower dose of **12** (50 mg kg^{-1}) for 16 h also resulted in a decrease in *O*-GlcNAc levels within multiple tissues ……………………………………………………………………………....S15-16

Figure S7. Lectin blotting does not reveal a change in extracellular glycans in response to *in vivo* exposure to **12**………………………………………………………………….S8

Figure S8. Mice exposed to 5SGlcNHex have large stomachs full of rodent chow………S9

Figure S9. 5SGlcNAc-OAc (**3**-OAc) and **12** reduce levels of *O*-GlcNAc-modified transcription factors known to regulate leptin production in adipocytes and *in vivo* ……………………………………………………………………..………..S13-14

Figure S10. A hypothesized link between the hexosamine biosynthetic pathway, OGT, and nutrient-sensing via leptin signaling…………………………………………………S10

Figure S11. Effects of Ac$_4$5SGlcNAc (**3**-OAc) in differentiated 3T3-L1 adipocytes ………………………………………………………………………………S11-12

Supplemental Methods

Synthesis of 5SGlcNR analogues……………………………………………………...S17
General procedures……………………………………………………………………S22
Capillary electrophoresis analysis of nucleotide sugars and sugar phosphates
In vitro analysis of 5SGlcNR processing by GlcNAc-salvage enzymes

Cell culture
Animal care
Preparation of mouse tissues for immunoblot analysis
Immunoblotting
Statistical analysis

Supplemental References……………………………………………………………………..S26

SUPPLEMENTAL FIGURES

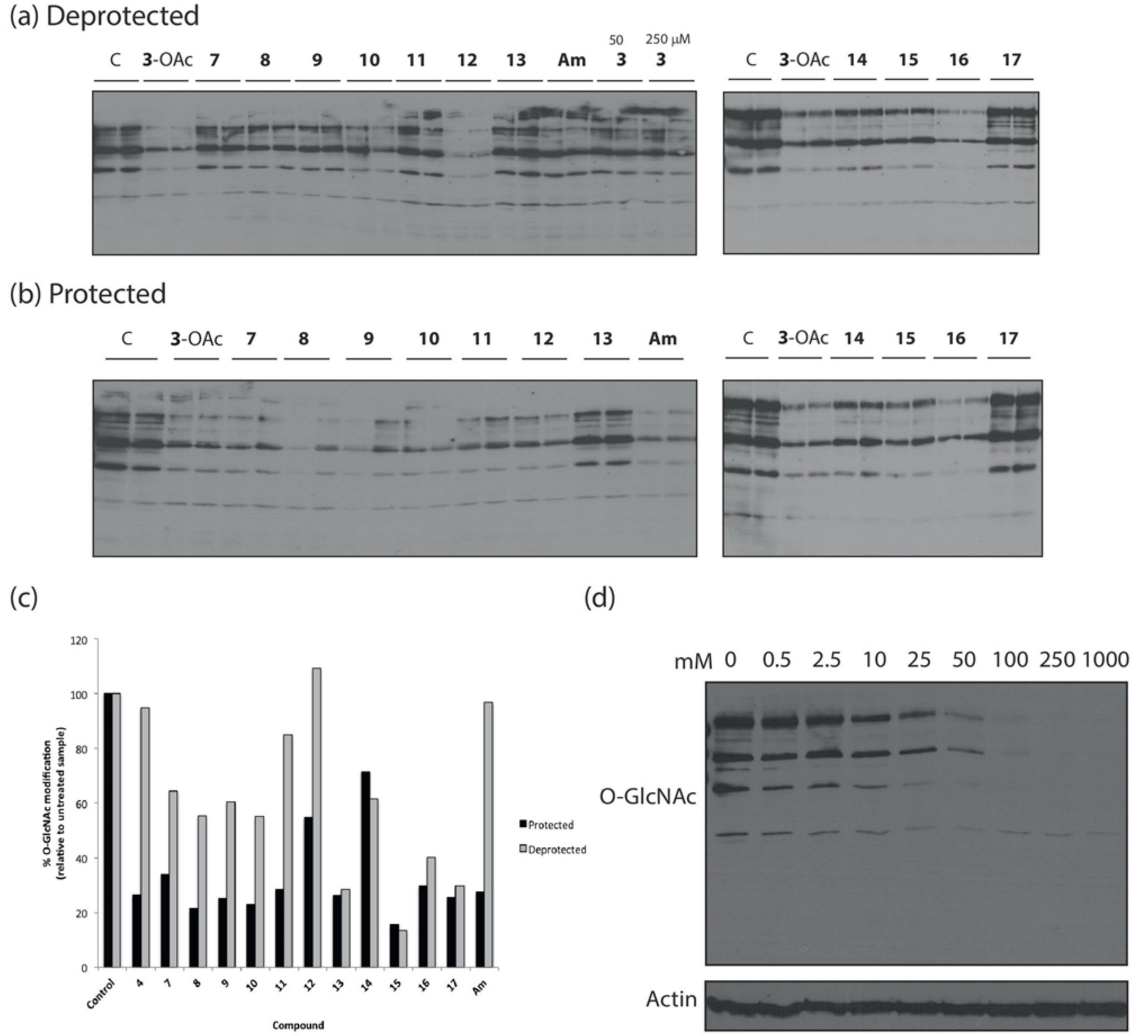

Figure S1. Comparison of protected and deprotected 5SGlcNR analogues (7-17) in HEK293 cells. a) Deprotected (i.e., de-*O*-acetylated) and **b)** protected 5SGlcNR analogues were added to subconfluent cells at a concentration of 50 µM for 24 h before cells were harvested. *O*-GlcNAc levels were assessed by immunoblot analysis. For both **a** and **b** the fluorescence signal obtained from the vehicle-treated control (C) cells was adjusted to 100 percent and all inhibitors tested were normalized to this. **c)** Relative *O*-GlcNAc levels were assessed by immunoblot analysis. **d)** Treating cells with 0 – 1,000 mM of **12** for 24 h showed a dose-dependent decrease *O*-GlcNAc levels by immunoblot analysis with the anti-*O*-GlcNAc antibody CTD110.6.

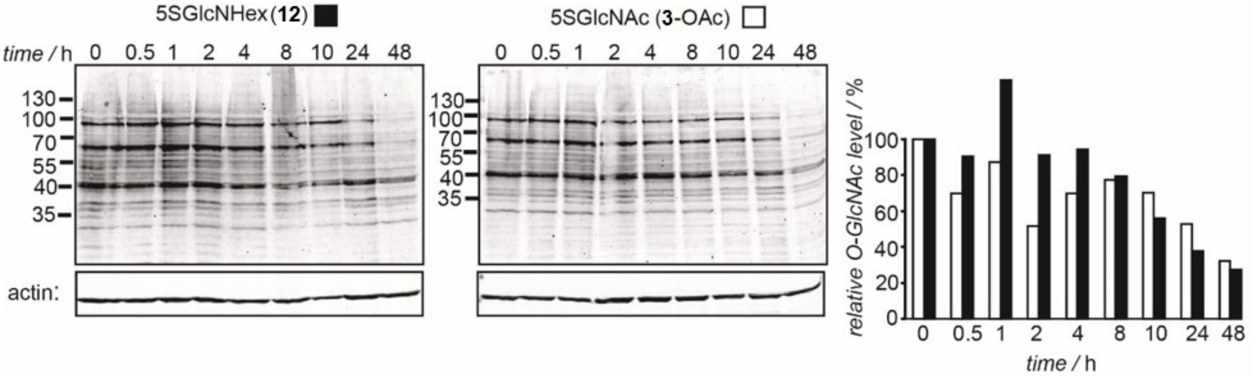

Figure S2. Time-course experiments in CHO K1 cells demonstrate that 5SGlcNHex (12) and peracetylated 5SGlcNAc (3-OAc) prevent OGT activity with similar kinetics. Deprotected 5SGlcNHex (**12**) and peracetylated 5SGlcNAc (**3**-OAc) were added to subconfluent COS-7 cells at a concentration of 50 μM before cells were harvested. Relative *O*-GlcNAc levels were assessed by immunoblot analysis.

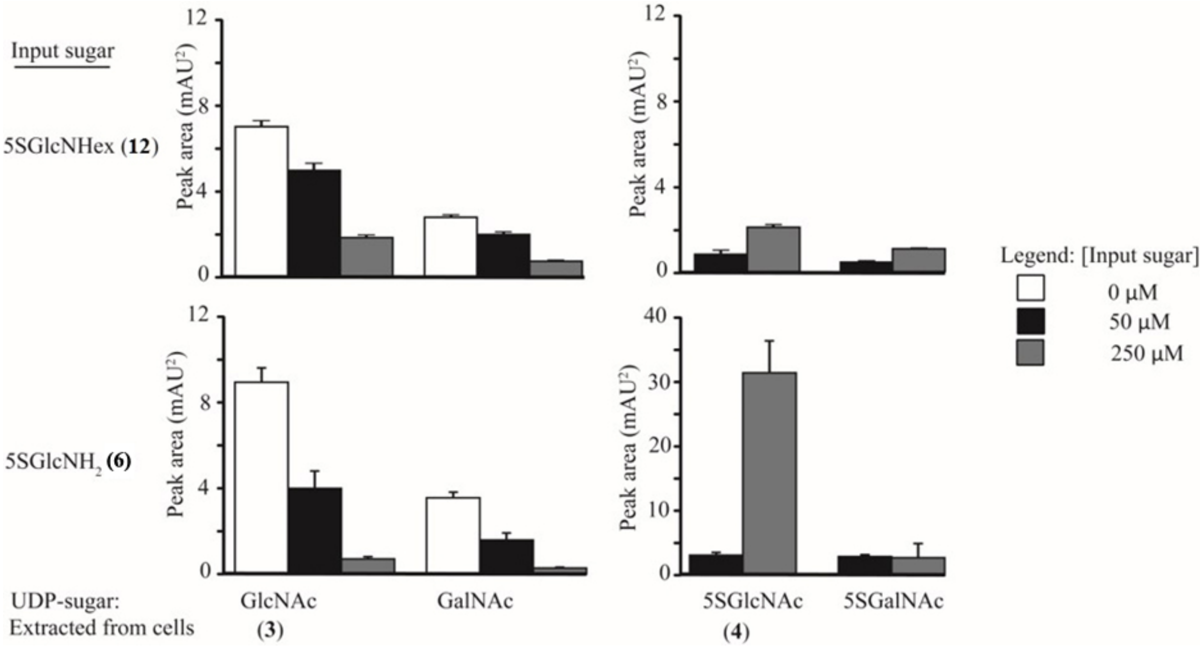

Figure S3. Quantification of nucleotide sugars extracted from 5SGlcNHex (12) and peracetylated 5SGlcNH₂ (6-OAc)-treated cells. COS7 cells were grown in the presence of increasing concentrations of **12** or **6**-OAc for 24 h after which their nucleotide sugar levels were extracted by solid-phase extraction and analyzed by CE. For each condition n = 3 and mean peak areas are reported ± the standard error of the mean. Notice that over this period much more UDP-5S-GlcNAc accumulated **6**-OAc-treated cells than in those exposed to **12**, although both compounds demonstrated the same level of feedback inhibition on UDP-GlcNAc biosynthesis.

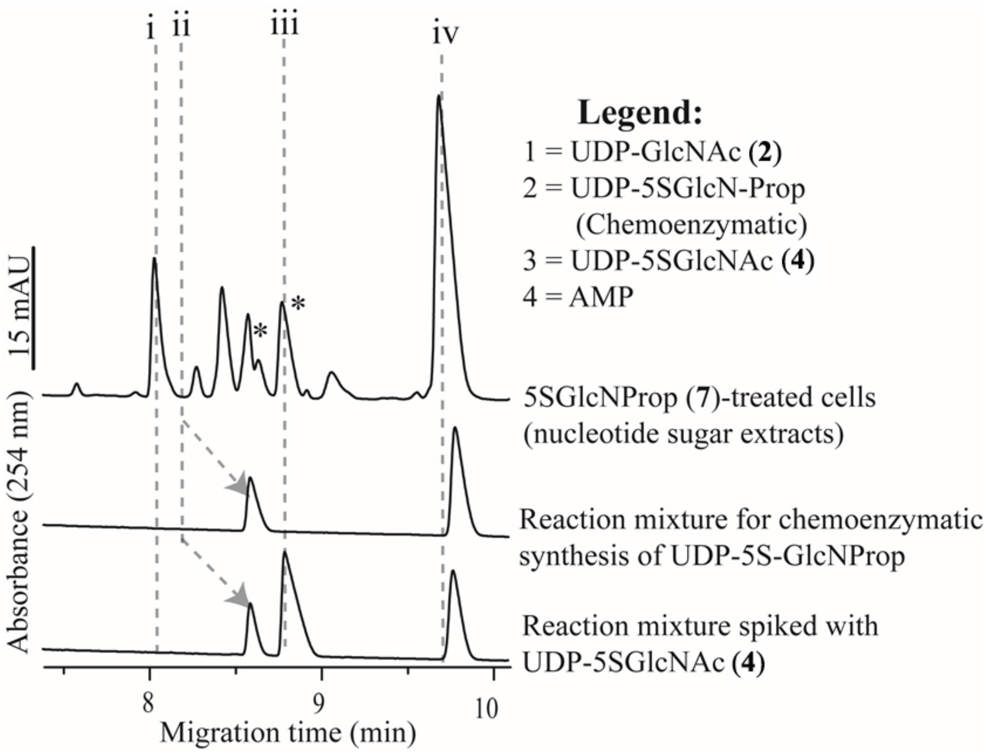

Figure S4. 5SGlcNPropyl (7) is a substrate for the enzymes of the GlcNAc-salvage pathway *in vitro* but not in cells. Nucleotide sugars from **7**-treated cells were extracted and analyzed by CE (top). Two new nucleotide sugars (these are both marked with a *) not observed in control cells were found in these extracts. One of these new nucleotide sugars (iii) had the same electrophoretic as chemoenzymatically-prepared UDP-5SGlcNAc (**4**; bottom). Significantly, the nucleotide sugar produced chemoenzymatically from **7** differs in mobility from both **4** prepared chemoenzymatically and the new nucleotide (*) produced in cells (middle). Notably, this chemoenzymatically prepared nucleotide sugar had an *m/z* $(M+H)^+$ = 680.5 (calc'd = 677.5); this ion was not present in the nucleotide sugar extracts obtained from cells although **4** could be detected. These data indicate that while UDP-5SGlcNProp may be produced in vitro since **7** is a substrate for the enzymes of the GlcNAc-salvage pathway, nevertheless, only **4** is produced in cells. This suggests that de-*N*-acetylation by NAGA is rapid relative to isomerisation by AGM.

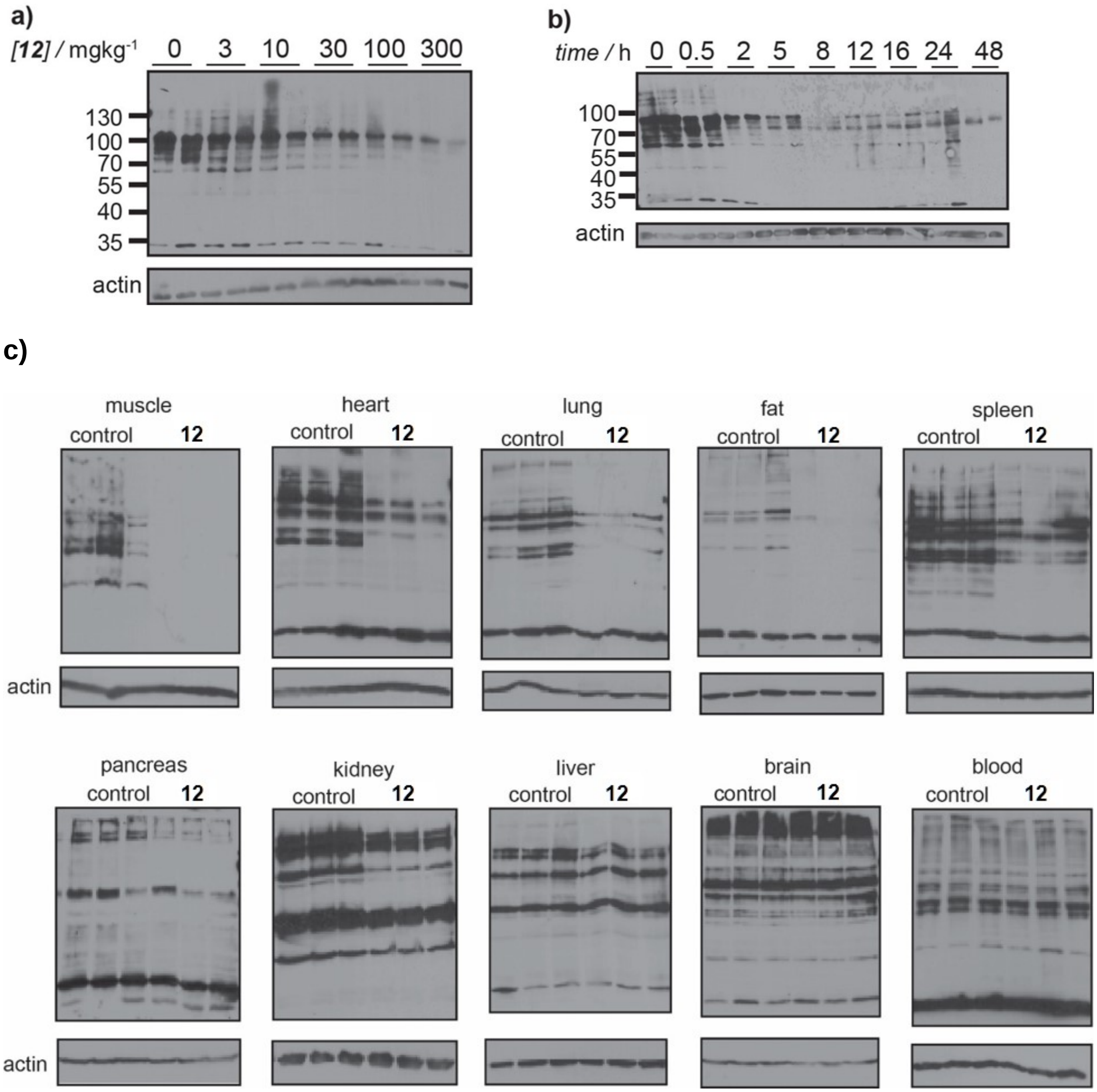

Figure S5. Exposure of mice to 5SGlcNHex (12) for 16 h resulted in the decrease in *O*-GlcNAc levels across multiple tissues. a) Treating mice with 0, 3, 30, 100, and 300 mg kg^{-1} of **12** showed a dose-dependent decrease spleen *O*-GlcNAc levels by immunoblot analysis with the anti-*O*-GlcNAc antibody CTD110.6. **b)** The onset of this decrease appeared as soon as 30 min after injection. **c)** Exposure of mice to 300 mg kg^{-1} of **12** for 16 h resulted in the decline in *O*-GlcNAc levels across multiple tissues.

a)

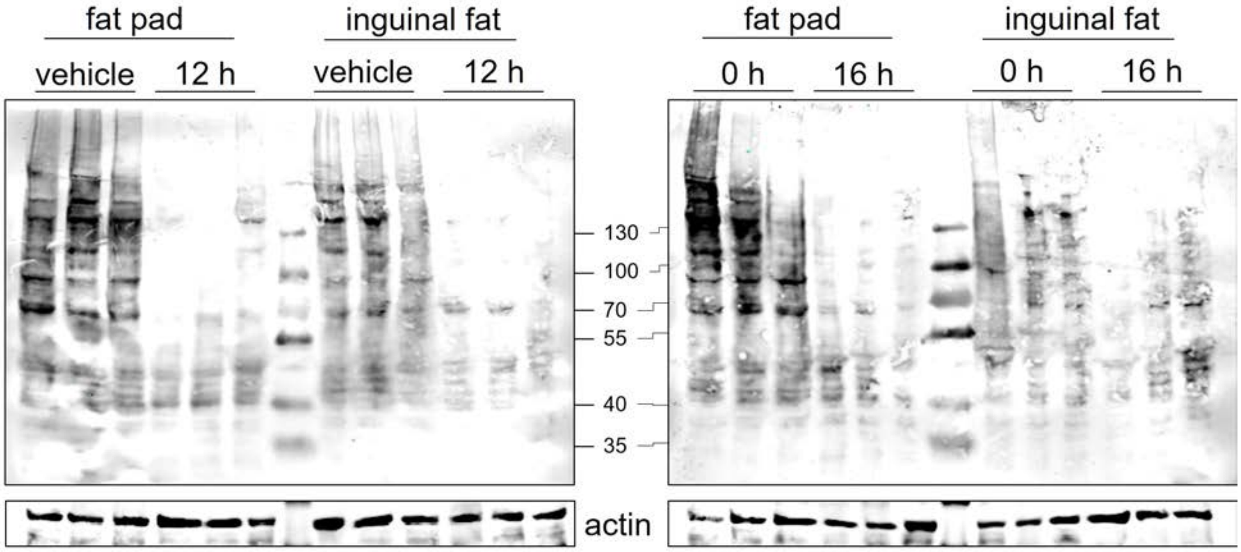
fat pad
vehicle
12 h
inguinal fat
vehicle
12 h
fat pad
0 h
16 h
inguinal fat
0 h
16 h
130
100
70
55
40
35
actin

b)

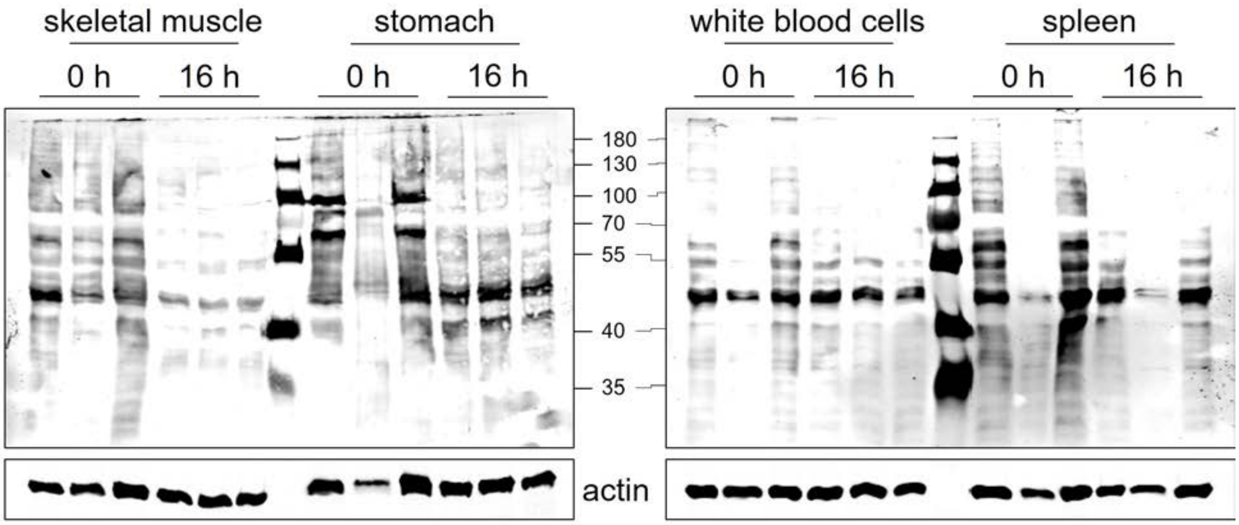
skeletal muscle
0 h
16 h
stomach
0 h
16 h
white blood cells
0 h
16 h
spleen
0 h
16 h
180
130
100
70
55
40
35
actin

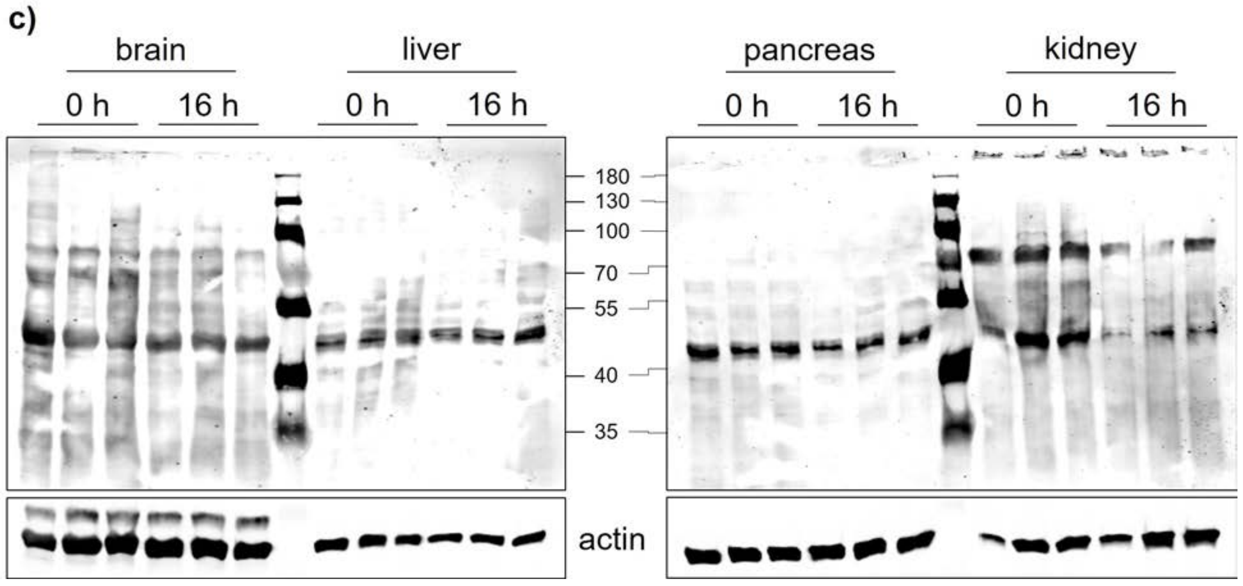

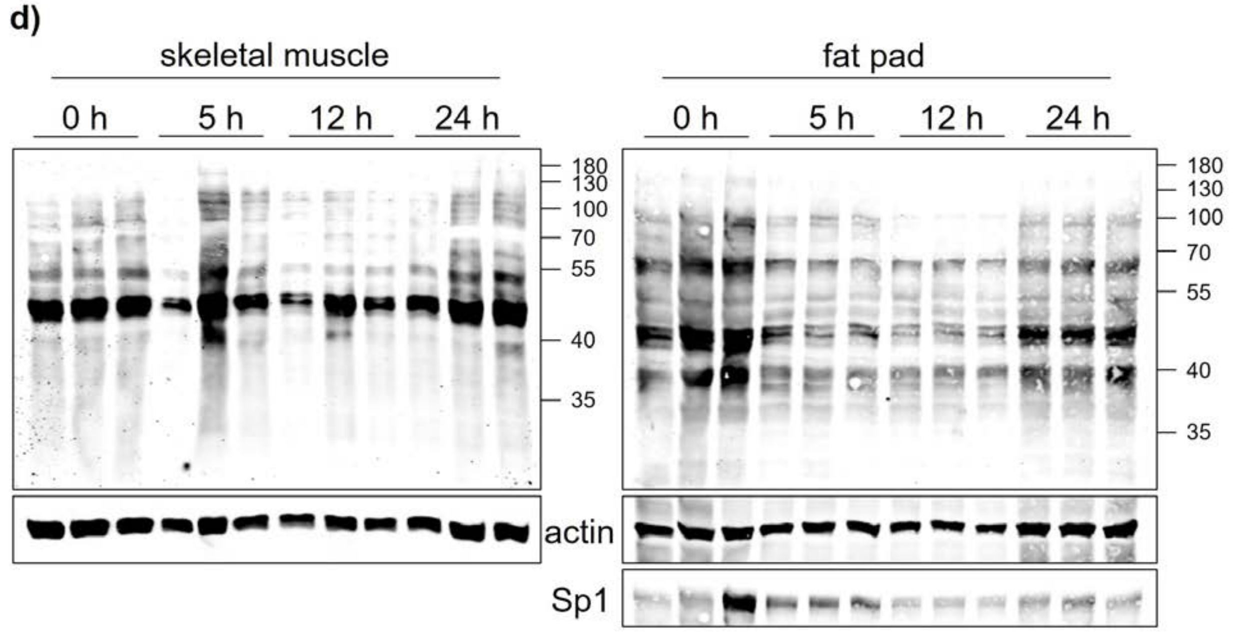

Figure S6. Exposure of mice to a lower dose of 12 (50 mg kg^{-1}) for 16 h also resulted in decreased *O*-GlcNAc levels in multiple tissues. Exposure of mice to 50 mg kg^{-1} of **12** for 16 h resulted in the decline in *O*-GlcNAc levels in **a)** inguinal fat, mammary fat pad, **b)** skeletal muscle, stomach, white blood cells, and spleen but not in the **c)** brain, liver, pancreas and kidney tissues. **d)** Treating mice with 50 mg kg^{-1} of **12** showed changes in skeletal muscle and fat pad *O*-GlcNAc and Sp1 levels by immunoblot analysis over time post-injection.

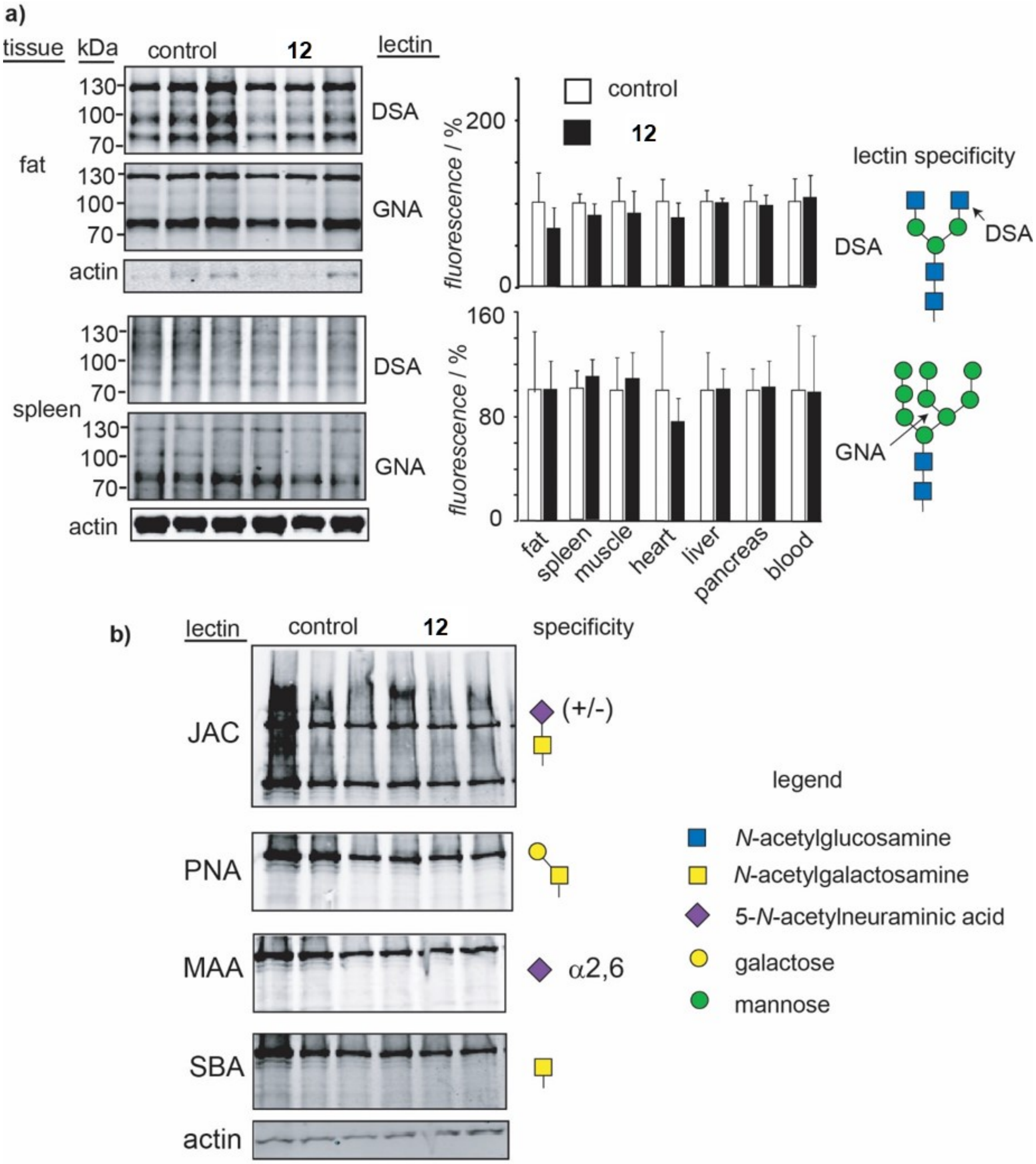

Figure S7. Lectin blotting does not reveal a change in extracellular glycans in response to *in vivo* exposure to 12. **a)** 5SGlcNHex (**12**) exposure did not significantly impact the biosynthesis of predominantly extracellular *N*-glycans in mouse tissues as assessed by lectin blotting. **b)** Lectin blots of mouse liver tissue with *O*-glycan-specific lectins likewise do not demonstrate any off-target effects of **12**.

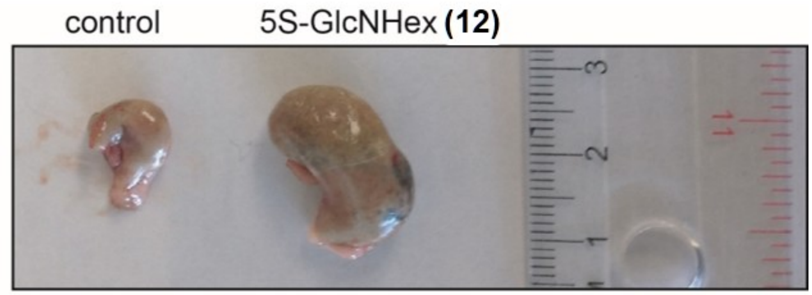

Figure S8. Mice exposed to 5SGlcNHex have large stomachs full of rodent chow. Photographs of stomachs removed from vehicle or 5SGlcNHex (**12**)-treated mice.

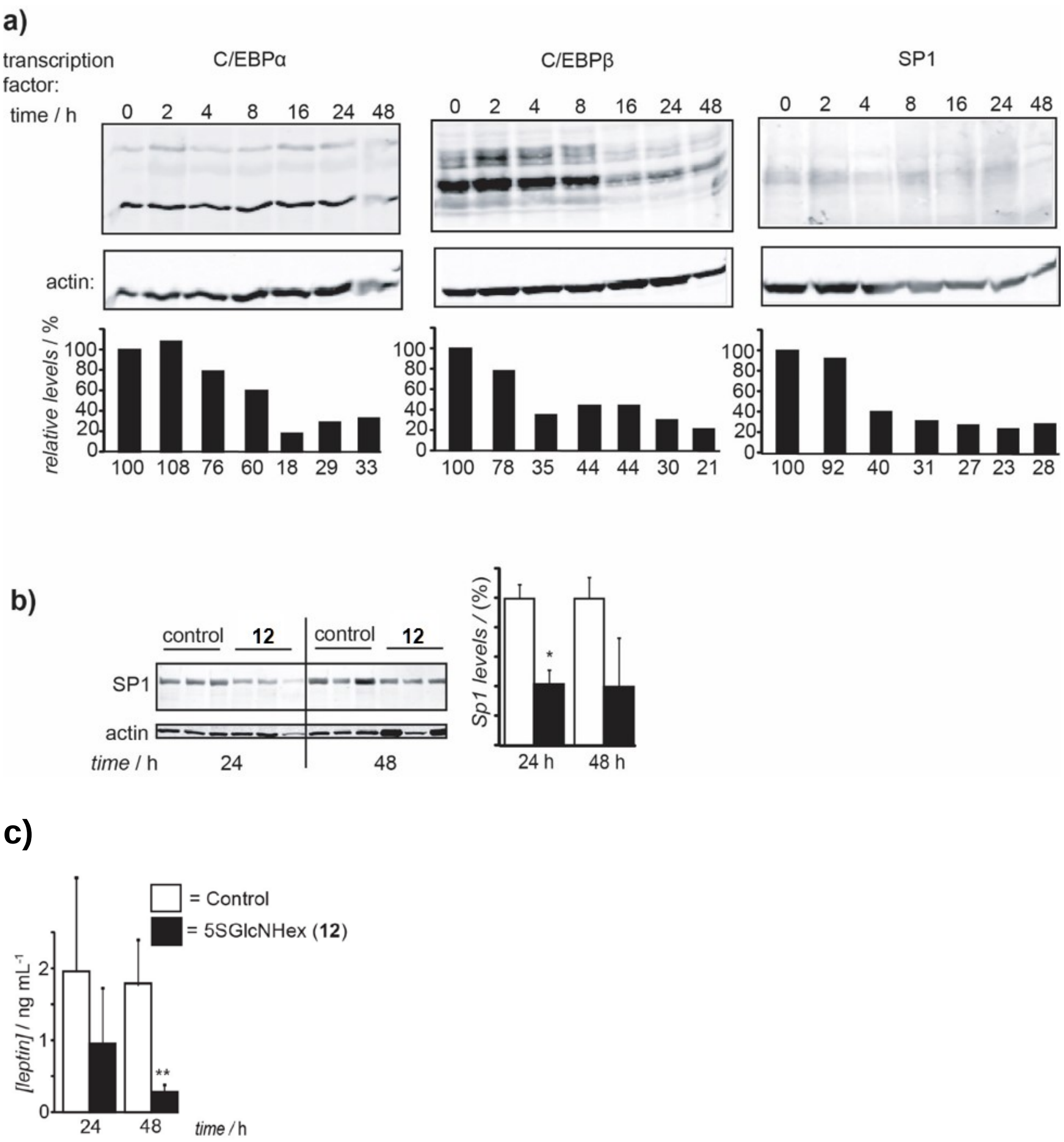

Figure S9. 5SGlcNAc-OAc (3-OAc) and 12 reduce levels of *O*-GlcNAc-modified transcription factors known to regulate leptin production in adipocytes and *in vivo*. a) Fully differentiated 3T3-L1 mouse adipocytes were grown in the presence of 5SGlcNHex **(12**) and harvested at the indicated time points. Immunoblot analyses using anti-C/EBP-α, -β, and anti-Sp1 monoclonal antibodies demonstrated that **12** induced the time-dependent decrease in all three transcription factors. All three transcription factors are known to be *O*-GlcNAc-modified, and all are known to regulate leptin transcription in adipocytes.[6,7] **b) 12** also led to a significant decrease in fat Sp1 levels *in vivo*. **c)** Serum analysis after a subsequent experiment demonstrated that **12** (300 mg kg^{-1}), significantly lowered leptin levels in treated mice. Mean Sp1 and leptin levels in adipose tissue were quantified 24 and 48 h after mice were treated with **12** and are reported ± SEM (n = 3 for both groups; *$p < 0.05$, **$p < 0.01$). At both time points, the values obtained for the control group were normalized to 100 percent (open bars).

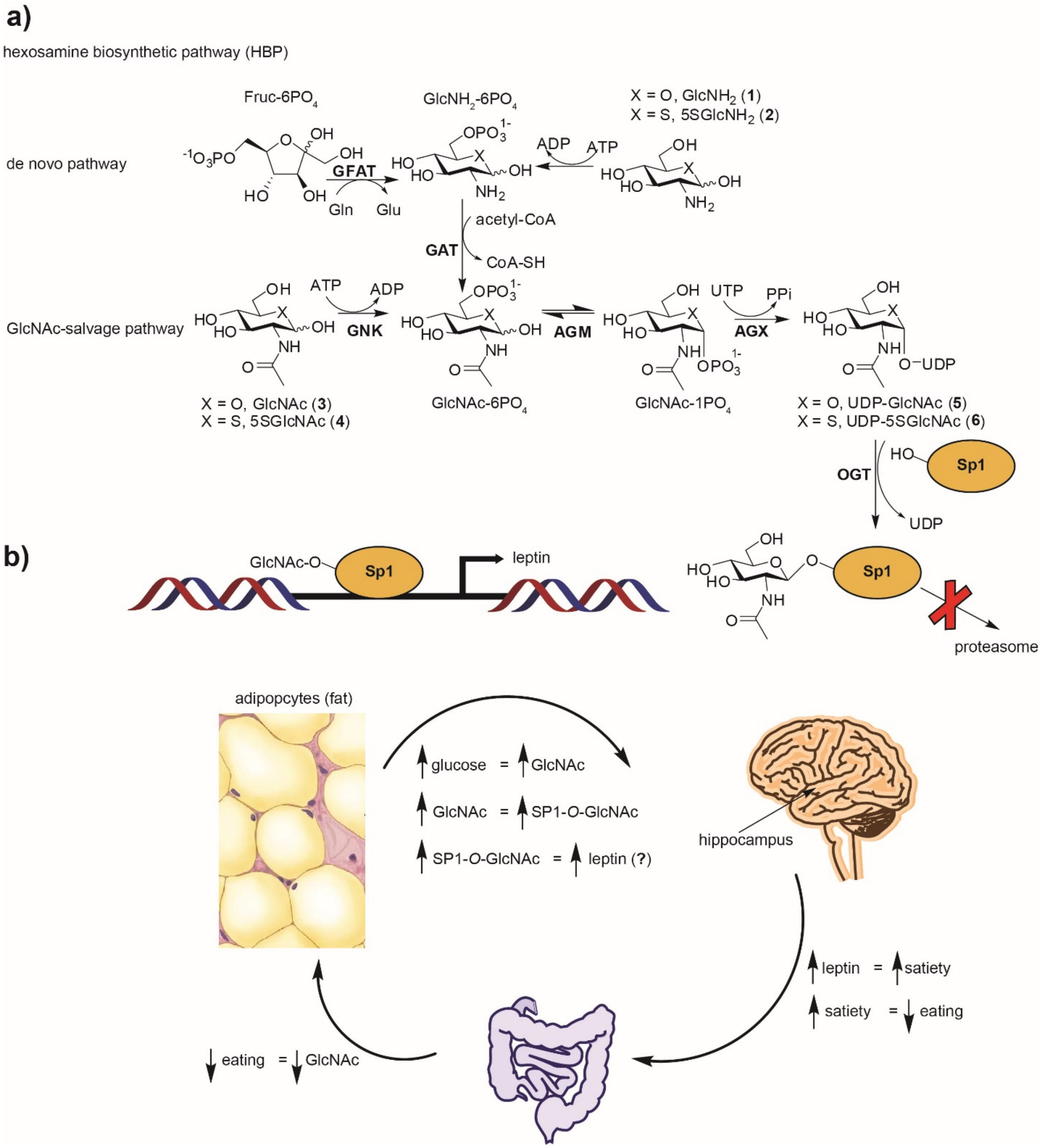

Figure S10. A hypothesized link between the hexosamine biosynthetic pathway, OGT, and nutrient-sensing via leptin signaling. a) Approximately five percent of all cellular glucose enters the HBP as fructose-6-phosphate (Fruc-6PO4) and ultimately gets biosynthesized into the OGT substrate UDP-GlcNAc (X = oxygen; **5**). We hypothesized that at high UDP-GlcNAc levels, such as would occur in fat or muscle tissue after a meal,[1] OGT would catalyze the *O*-GlcNAcylation of transcription factors such as Sp1 at a higher rate.[2–4] As *O*-GlcNAc-modified Sp1 is known to be protected from proteasomal degradation[5] increased Sp1 levels might **b)** lead to the increased transcription of the leptin gene in adipose (fat) tissue thereby inducing satiety and increasing energy expenditure.

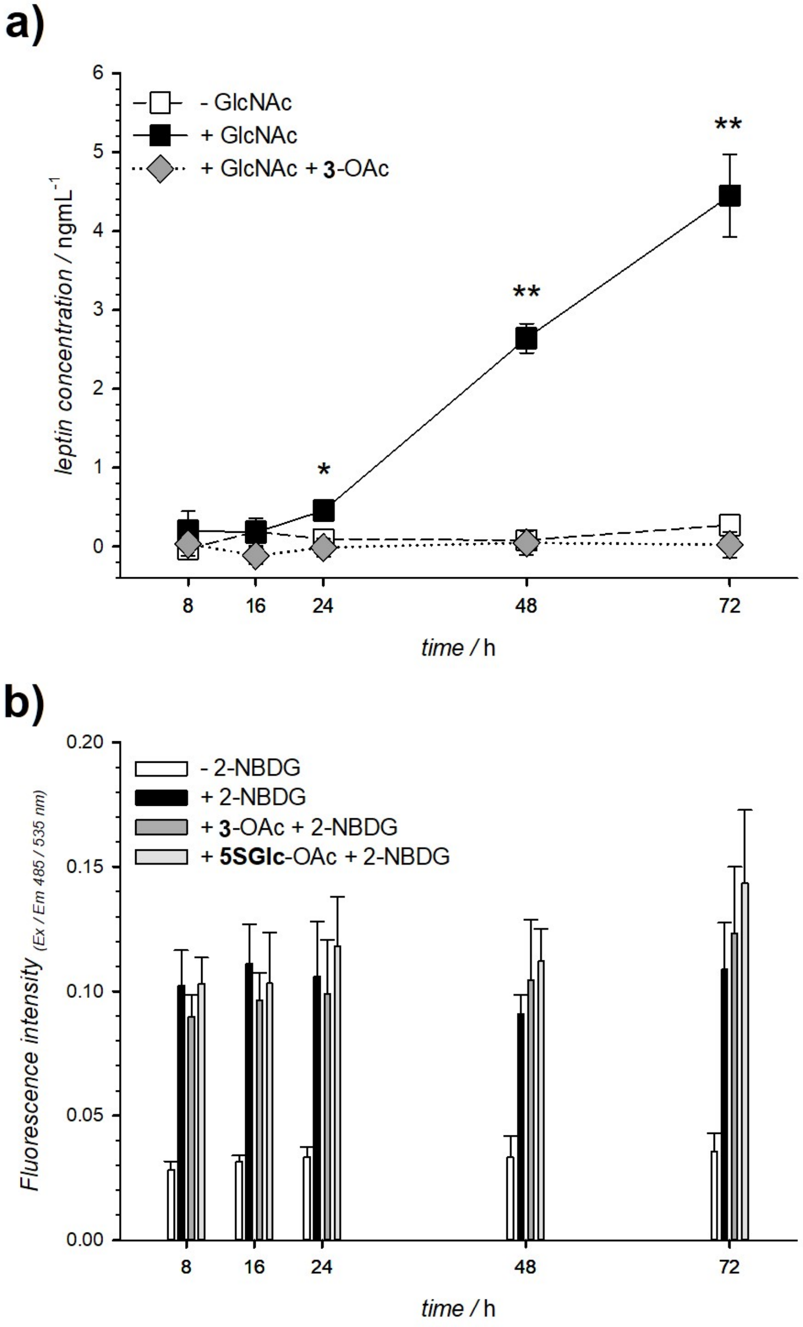

Figure S11. Effects of Ac$_4$5SGlcNAc (3-OAc) in differentiated 3T3-L1 adipocytes. a) 3**-OAc reduces GlcNAc-induced leptin secretion within 3T3-L1 adipocytes (n = 3 for each time point; *p<0.05, **p<0.01). **b) 2-NBDG uptake in 3T3-L1 adipocytes treated with **3**-OAc (deep gray) and peracetylated glucosyltransferase inhibitor, 5SGlucose (5SGlc-OAc, light gray). Treatment of 3T3-L1 adipocytes with **3**-OAc or 5SGlc-OAc did not significantly affect 2-NBDG uptake as compared to vehicle treated cells (white bars without 2-NBDG and black bars with 2-NBDG) showing that cells do not have altered viability or that the compounds used at these concentrations do not inhibit glucose transport.

SUPPLEMENTAL METHODS

Synthesis of 5SGlcNR analogues

General—Solvents were purchased from Sigma, Anachemia or Fisher and were ACS reagent grade or better unless otherwise indicated. Synthetic reactions were monitored by thin layer chromatography (TCL) using Merck (White House Station, NJ) Kiesselgel 60 F254 aluminum-backed sheets. After their separation compounds were detected on TLC plates by visualizing under ultraviolet light or by charring with 2 M H_2SO_4 in ethanol. All intermediate compounds were purified by flash column chromatography on silica gel (230-400 mesh, Merck Kieselgel) using the specified solvents. ^{1}H-NMR spectra were recorded on Bruker AVANCE 400, 500 or 600 MHz instruments as indicated. High-resolution mass spectra were obtained by the electrospray ionization method using an Agilent 6210 TOF LC/MS high-resolution magnetic sector mass spectrometer.

*Synthesis of N-acyl-α-D-5-thio-glucosamine (**7-17**) and N-acyl-1,3,4,6-tetra-O-acetyl-α-D-5-thio-glucosamine derivatives*—*N*-acetyl-α-D-5-thio-glucosamine (**3**) and its peracetylated analogue (**3-OAc)** were synthesized from *N*-acetyl-D-glucosamine precisely as previously described.[8] All *N*-acyl derivatives of **3**, and their peracetylated analogues, were synthesized as described in general procedures A and B, respectively, and outlined in Supplemental Scheme S1. General procedure A: Acid anhydrides (1.5 eq.) and Et_3N (1.1 eq.) were added into a solution of α-D-5-thio-glucosamine hydrochloride (**2**) dissolved in a mixture of H_2O and EtOH (1:1, v/v). The reaction mixture was stirred at 20 °C for 18-20 h. The solvent was then removed *in vacuo*, and the desired materials were isolated as solids by flash chromatography on silica using a solvent system of EtOAc:MeOH:H_2O in ratios ranging from 15:1:0.5 to 10:1:0.5 as appropriate. General procedure B: N-acyl-α-D-5-thio-glucosamine (5SGlcNR) analogues were dissolved in pyridine and Ac_2O (10 eq.) was added at 20 °C. The resulting reaction mixture was stirred for 18-20 h. The solvent was removed *in vacuo*, and the desired materials were isolated as solids by flash chromatography on silica gel using a solvent system of Hex:EtOAc in ratios ranging from 3:1 to 1 : 1 as appropriate.

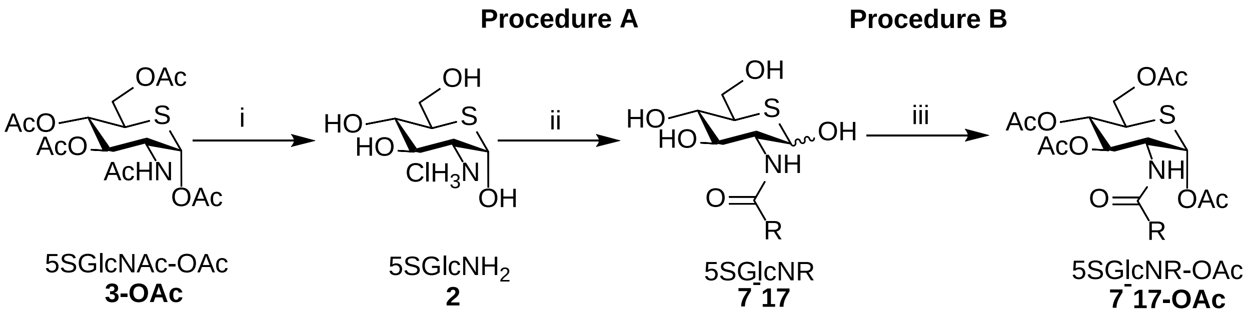

Supplementary scheme 1. Synthesis of 5SGlcNR analogues. i. 2N HCl, reflux, 4h; ii. $(RCO)_2O$, H_2SO_4, 20 °C; iii. Ac_2O, pyridine, 20 °C.

Synthesis of α-D-5-thio-glucosamine (5SGlcNH$_2$*, **2**) and 1,3,4,6-tetra-O-acetyl-*α-D-5-thio-glucosamine (**6-OAc**)—*N*-acetyl-1,3,4,6-tetra-*O*-acetyl-α-D-5-thio-glucosamine (**3-OAc**) (1.945 mg, 2.33 mmol) was suspended in 2 N HC1 (aq., 15 mL), and the resultant reaction mixture was stirred under reflux for 4 h. After cooling down to 20 °C the reaction mixture was concentrated

under high vacuum, and was co-evaporated with toluene (8 x 15 m). The residue was re-dissolved in MeOH (100 mL), and activated carbon was added to the solution. After stirring for 30 min, the mixture was filtered through Celite, and concentrated to dryness. The residue was further dried under high vacuum to afford **6** as a solid (520 mg, 96%), which was used directly to the next reaction without purification. ^{1}H-NMR (500 MHz, D_2O): δ (ppm) 5.23 (d, *J* = 3.00 Hz, 1 H), 3.94 (dd, *J* = 12.0, 5.50 Hz, 1 H), 3.89 (dd, *J* = 12.0, 3.00 Hz, 1 H), 3.84 (dd, *J* = 10.0, 9.00 Hz, 1 H), 3.67 (dd, *J* = 10.5, 9.00 Hz, 1 H), 3.61 (dd, *J* = 10.5, 3.00 Hz, 1 H), 3.30 (ddd, *J* = 10.0, 5.50, 3.00 Hz, 1 H). ^{13}C-NMR (125 MHz, D_2O): δ (ppm) 73.98, 71.16, 70.51 , 60.45, 59.06, 43.84. HRMS *(m/z)*: $C_6H_{14}NO_4S$ $(M+H)^+$: Calc'd, 196.0638; Found, 196.0634. $C_6H_{13}NNaO_4S$ $(M+Na)^+$: Calc'd, 218.0457; Found, 218.0457. The per-*O*-acetylated sulfuric acid salt of **6** was prepared by dissolving **6** (20.0 mg, 0.086 mmol) in Ac_2O (0.30 mL) and adding and concentrated H_2SO_4 (1 drop, about 0.025 mL). The resulting mixture was stirred at 20 ^{o}C for 40 h. After the addition of MeOH (2 drops), the mixture became clear. The reaction mixture was stirred on an ice bath while EtOAc (about 3.0 mL) was added. A white solid was precipitated; this was filtered and washed with EtOAc (20 mL) to afford **6-OAc** as a white solid. This was purified by recrystallization from EtOAc and MeOH. The yield was 10 mg (25 %). ^{1}H-NMR (400 MHz, MeOD): δ (ppm) 6.12 (d, *J* = 3.20 Hz, 1 H), 5.40 (t, *J* = 10.0 Hz, 1 H), 5.31 (t, *J* = 10.0 Hz, 1 H), 4.46 (dd, *J* = 12.4, 4.80 Hz, 1 H), 4.12 (dd, *J* = 10.8, 3.20 Hz, 1), 4.04 (dd, *J* = 12.4, 3.20 Hz, 1 H), 3.72 (ddd, *J* = 10.8, 4.40, 3.20 Hz, 1 H), 2.23 (s, 3 H), 2.12 (s, 3 H), 2.05 (s, 3 H), 2.02 (s, 3 H). ^{13}C-NMR (100 MHz, MeOD): δ (ppm) 172.01, 171.58, 171.16, 170.17, 73.03, 72.10, 71.96, 62.01, 56.88, 40.84, 20.82, 20.7, 20.51, 20.48. HRMS *(m/z)*: $C_{14}H_{22}NO_8S$ $(M+H)^+$: Calc'd, 364.1061; Found, 364.1068. $C_{14}H_{21}NNaO_8S$ $(M+Na)^+$: Calc'd, 386.0880; Found, 386.0890.

*Synthesis of N-proprionyl-α-D-5-thio-glucosamine (**7**) and N-proprionyl-1,3,4,6-tetra-O-acetyl- α-D-5-thio-glucosamine (**7-OAc**)*—These compounds were prepared from **6** following general procedures A and B. For **7**: ^{1}H-NMR (400 MHz, D_2O): δ (ppm) 4.99 (d, 7 = 2.80 Hz, 1 H, H-l), 4.11 (dd, *J* = 10.4, 2.80 Hz, 1 H, H-2), 3.95 (dd, *J* = 12.0, 5.60 Hz, 1 H, H-6a), 3.89 (dd, *J*= 12.0, 3.20 Hz, 1 H, H-6b), 3.73 (t, *J*= 9.20 Hz, 1 H, H- 3), 3.68 (t, *J*= 9.20 Hz, 1 H, H-4), 3.28 (ddd, *J*= 9.60, 5.60, 3.60 Hz, 1 H, H-5), 2.31 (q, *J* = 7.60 Hz, 2 H, H-2'), 1.13 (t, *J*= 7.60 Hz, 3 H, H-3'). ^{13}C-NMR (100 MHz, D_2O): δ (ppm) 178.00 (C-1'), 74.04 (C-4), 71.75 (C-l), 71.52 (C-3), 60.20 (C-6), 58.05 (C- 2), 43.15 (C-5), 29.08 (C-2'), 9.49 (C-3'). HRMS *(m/z)*: $C_9H_{17}NO_5S$ $(M+H)^+$: Calc'd, 252.0900; Found, 252.0895. $C_9H_{16}NO_5S$ $(M+Na)^+$: Calc'd, 274.0720; Found, 274.0718. For **7-OAc**: ^{1}H-NMR (500 MHz, $CDCl_3$): δ (ppm) 5.93 (d, *J* = 3.00 Hz, 1 H), 5.71 (d, *J* = 9.00 Hz, 1 H), 5.37 (t, *J* = 10.0 Hz, 1 H), 5.18 (t, *J* = 10.5 Hz, 1 H), 4.66-4.61 (m, 1 H), 4.32 (dd, *J*= 12.0, 5.00 Hz), 4.03 (dd, *J* = 12.0, 3.00 Hz), 3.46 (ddd, *J* = 11.0, 5.00, 3.50 Hz, 1 H), 2.17 (s, 3 H), 2.15-2.05 (m, 2 H), 2.06 (s, 3 H), 2.03 (s, 3 H), 2.01 (s, 3 H), 1.06 (t, *J* = 7.50 Hz, 3 H). ^{13}C-NMR (125 MHz, $CDC1_3$): δ (ppm) 173.21, 171.57, 170.51, 169.08, 168.68, 72.69, 71.69, 71.43, 61.07, 54.99, 39.70, 29.50, 21.03, 20.58, 20.46, 9.63. HRMS (*m/z*): $C_7H_{26}NO_9S$ $(M+H)^+$: Calc'd, 420.1323; Found, 420.1324.

*Synthesis of N-(n-butyryl)-a-D-5-thio-glucosamine (**8**) and N-(n-butyryl)-l,3,4,6-tetra-O-acetyl- α-D-5-thio-glucosamine (**8-OAc**)*—These compounds were prepared from **6** following general procedures A and B. For **8**: ^{1}H-NMR (500 MHz, D_2O): δ (ppm) 4.97 (d, *J* = 3.00 Hz, 1 H), 4.10 (dd, 7 = 10.5, 3.00 Hz, 1 H), 3.93 (dd, *J* = 12.0, 5.50 Hz, 1 H), 3.89 (dd, *J* = 12.0, 3.50 Hz, 1 H), 3.70 (t, *J* = 9.50 Hz, 1 H), 3.66 (t, *J* = 9.50 Hz, 1 H), 3.27 (ddd, *J* = 10.0, 5.50, 3.00 Hz, 1 H), 2.26 (t, *J* = 7.50 Hz, 2 H), 1.65-1.57 (m, 2 H), 0.90 (t, *J* = 7.50 Hz, 3 H). ^{13}C-NMR (125 MHz, D_2O): δ (ppm) 177.07, 74.02, 71.79, 71.41, 60.15, 58.02, 43.11, 37.56, 18.99, 12.63. HRMS (*m/z*):

$C_{10}H_{20}NO_5S$ $(M+H)^+$: Calc'd, 266.1057; Found, 266.1052. $C_{10}H_{19}NNaO_5S$ $(M+Na)^+$: Calc'd, 288.0876; Found, 288.0876. For **8-OAc**: ^{1}H-NMR (500 MHz, $CDCl_3$): δ (ppm) 5.94 (d, *J* = 3.00 Hz, 1 H), 5.71 (d, *J* = 9.00 Hz, 1 H), 5.37 (t, *J* = 10.0 Hz, 1 H), 5.18 (t, *J* = 10.0 Hz, 1 H), 4.66-4.61 (m, 1 H), 4.32 (dd, *J* = 12.5, 5.00 Hz, 1 H), 4.03 (dd, *J* = 12.0, 3.00 Hz, 1 H), 3.47 (ddd, *J* = 11.0, 5.00, 3.50 Hz, 1 H), 2.17 (s, 3 H), 2.10-2.06 (m, 2 H), 2.06 (s, 3 H), 2.03 (s, 3 H), 2.02 (s, 3 H), 1.60-1.53 (m, 2 H), 0.88 (t, *J* = 7.50 Hz, 3 H. ^{13} C- NMR (125 MHz, $CDCl_3$): δ (ppm) 172.39, 171.63, 170.51, 169.11, 168.66, 72.69, 71.64, 71.46, 61.08, 55.01, 39.70, 38.71, 21.04, 20.64, 20.60, 20.49, 18.94, 13.51. HRMS *(m/z)*: $C_{18}H_{28}NO_9S$ $(M+H)^+$: Calc'd, 434.1479; Found, 434.1484. $C_{18}H_{27}NNaO_9S$ $(M+Na)^+$: Calc'd, 456.1299; Found, 456.1304.

*Synthesis of N-(i-butyryl)-a-D-5-thio-glucosamine (**9**) and N-(i-butyryl)-l,3,4,6-tetra-O-acetyl- α-D-5-thio-glucosamine (**9-OAc**)*—These compounds were prepared from **6** following general procedures A and B. For **9**: ^{1}H-NMR (400 MHz, D_2O): δ (ppm) 4.98 (d, *J*= 2.80 Hz, 1 H), 4.12 (dd, *J* = 10.4, 2.80 Hz, 1 H), 3.94 (dd, *J* = 11.6, 5.60 Hz, 1 H), 3.90 (dd, *J* = 12.0, 3.20 Hz, 1 H), 3.73 (t, *J* = 9.60 Hz, 1 H), 3.68 (t, *J*= 9.60 Hz, 1 H), 3.29 (ddd, *J* = 9.60, 5.60, 3.20 Hz, 1 H), 2.63-2.52 (m, 1 H), 1.12 (d, *J* = 6.80 Hz, 6 H). ^{13}C-NMR (100 MHz, D_2O): 181.11, 74.07, 71.80, 71.48, 60.22, 57.93, 43.17, 34.97, 18.72, 18.55. HRMS (*m/z*): $C_{10}H_{20}NO_5S$ $(M+H)^+$: Calc'd, 266.1057; Found, 266.1052. $C_{10}H_{19}NNaO_5S$ $(M+Na)^+$: Calc'd, 288.0876; Found, 288.0874. For **9-OAc**: ^{1}H-NMR (500 MHz, $CDCl_3$): δ (ppm) 5.95 (d, *J* = 3.00 Hz, 1 H), 5.69 (d, *J* = 9.00 Hz, 1 H), 5.38 (t, *J*= 10.0 Hz, 1 H), 5.18 (t, *J* = 10.0 Hz, 1 H), 4.65- 4.59 (m, 1 H), 4.34 (dd, *J* = 12.0, 5.00 Hz, 1 H), 4.04 (dd, *J* = 12.0, 3.00 Hz, 1 H), 3.48 (ddd, *J* = 11.0, 5.00, 3.50 Hz, 1 H), 2.30-2.21 (m, 1 H), 2.18 (s, 3 H), 2.06 (s, 3 H), 2.04 (s, 3 H), 2.02 (s, 3 H), 1.06 (d, *J* = 5.00 Hz, 3 H), 1.05 (d, *J* = 5.00 Hz, 3 H). ^{13}C-NMR (125 MHz, $CDC1_3$): δ (ppm) 176.32, 171.63, 170.54, 169.12, 168.65, 72.61, 71.66, 71.44, 61.10, 54.93, 39.73, 35.41, 21.00, 20.62, 20.49, 19.38, 19.17. HRMS (mlz): $C_{18}H_{28}NO_9S$ $(M+H)^+$: Calc'd, 434.1479; Found, 434.1482. $C_{18}H_{27}NNaO_9S$ $(M+Na)^+$: Calc'd, 456.1299; Found, 456.1300.

*Synthesis of N-(n-valetyl)-a-D-5-thio-glucosamine (**10**) and N-(n-valetyl)-l,3,4,6-tetra-O-acetyl-α-D-5-thio-glucosamine (**10-OAc**)*—These compounds were prepared from **6** following general procedures A and B. For **10**: ^{1}H-NMR (500 MHz, D_2O): δ (ppm) 4.96 (d, *J* = 2.50 Hz, 1 H), 4.10 (dd, *J* = 10.0, 3.00 Hz, 1 H), 3.93 (dd, *J* = 12.0, 5.50 Hz, 1 H), 3.87 (dd, *J* = 12.0, 3.00 Hz, 1 H), 3.70 (t, *J* = 9.50 Hz, 1 H), 3.66 (t, *J* = 10.0 Hz, 1 H), 3.27 (ddd, *J* = 9.50, 5.50, 3.00 Hz, 1 H), 2.29 (t, *J* = 7.50 Hz, 2 H), 1.60-1.54 (m, 2 H), 1.34-1.27 (m, 2 H), 0.88 (t, *J* = 7.50 Hz, 3 H). ^{13}C-NMR (125 MHz, D_2O): δ (ppm) 177.19, 73.88, 71.66, 71.30, 60.00, 57.93, 43.00, 35.34, 27.47, 21.40, 12.90. HRMS *(m/z)*: $C_{11}H_{22}NO_5S$ $(M+H)^+$: Calc'd, 280.1213; Found, 280.1200. $C_{11}H_{21}NNaO_5S$ $(M+Na)^+$: Calc'd, 302.1033; Found, 302.1023. For **10-OAc**: ^{1}H-NMR (500 MHz, $CDCl_3$): δ (ppm) 5.93 (d, *J*= 3.50 Hz, 1 H), 5.71 (d, *J*= 8.50 Hz, 1 H), 5.38 (dd, *J*= 11.0, 10.0 Hz, 1 H), 5.18 (dd, *J*= 11.0, 10.0 Hz, 1 H), 4.65-4.60 (m, 1 H), 4.34 (dd, *J*= 12.0, 5.00 Hz, 1 H), 4.02 (dd, *J*= 12.0, 3.50 Hz, 1 H), 3.47 (ddd, *J*= 10.5, 4.50, 3.00 Hz, 1 H), 2.17 (s, 3 H), 2.10-2.05 (m, 2 H), 2.06 (s, 3 H), 2.04 (s, 3 H), 2.02 (s, 3 H), 1.53-1.47 (m, 2 H), 1.29-1.22 (m, 2 H), 0.87 (t, *J*= 7.50 Hz, 3 H). ^{13}C-NMR (125 MHz, CDCI3): δ (ppm) 172.56, 171.57, 170.50, 169.09, 168.63, 72.66, 71.62, 71.46, 61.07, 54.99, 39.68, 36.18, 27.53, 22.12, 21.00, 20.59, 20.57, 20.46, 13.63. HRMS *(m/z)*: $C_9H_{30}NO_9S$ $(M+H)^+$: Calc'd, 448.1636; Found, 448.1640. $C_{19}H_{29}NNaO_9S$ $(M+Na)^+$: Calc'd, 470.1455; Found, 470.1459.

*Synthesis of N-(n-valeryl)-a-D-5-thio-glucosamine (**11**) and N-(n-valeryl)-l,3,4,6-tetra-O-acetyl-α-D-5-thio-glucosamine (**11-OAc**)*—These compounds were prepared from **6** following general

procedures A and B. For **11**: ^{1}H-NMR (500 MHz, D_2O): δ (ppm) 4.97 (d, *J* = 2.50 Hz, 1 H), 4.10 (dd, 7 = 10.0, 3.00 Hz, 1 H), 3.93 (dd, 7 = 12.0, 5.50 Hz, 1 H), 3.90 (dd, *J* = 12.0, 3.50 Hz, 1 H), 3.70 (t, *J* = 9.50 Hz, 1 H), 3.66 (t, *J* = 9.50 Hz, 1 H), 3.26 (ddd, *J* = 9.50, 5.00, 3.00 Hz, 1 H), 2.15 (d, *J* = 7.50 Hz, 2 H), 2.03-1.95 (m, 1 H), 0.92 (d, 7 = 6.50 Hz, 3 H), 0.91 (d, *J* = 6.50 Hz, 3 H). ^{13}C-NMR (125 MHz, D_2O): δ (ppm) 176.45, 74.06, 71.84, 71.38, 60.16, 58.01, 44.86, 43.12, 26.20, 21.54, 21.36. HRMS *(m/z)*: $C_{11}H_{22}NO_5S$ $(M+H)^+$: Calc'd, 280.1213; Found, 280.1201. $C_{11}H_{21}NNaO_5S$ $(M+Na)^+$: Calc'd, 302.1033; Found, 302.1022. For **11-OAc**: ^{1}H-NMR (500 MHz, CDCl3): δ (ppm) 5.95 (d, *J* = 3.00 Hz, 1 H), 5.71 (d, *J* = 8.50 Hz, 1 H), 5.37 (dd, *J* = 10.5, 9.50 Hz, 1 H), 5.18 (dd, *J* = 11.0, 10.0 Hz, 1 H), 4.65-4.60 (m, 1 H), 4.34 (dd, *J* = 12.0, 5.00 Hz, 1 H), 4.03 (dd, 7 = 12.0, 3.00 Hz, 1 H), 3.47 (ddd, *J*= 11.0, 5.00, 3.00 Hz, 1 H), 2.16 (s, 3 H), 2.06 (s, 3 H), 2.04 (s, 3 H), 2.02 (s, 3 H), 2.01-1.92 (m, 3 H), 0.87 (d, *J*= 6.50 Hz, 6 H). ^{13}C-NMR (125 MHz, $CDCl_3$): (S 171.88, 171.66, 170.52, 169.1 1, 168.62, 72.64, 71.56, 71.50, 61.08, 55.07, 45.77, 39.69, 26.02, 22.20, 22.1 1, 21.01, 20.67, 20.60, 20.48. HRMS *(m/z)*: $C_{19}H_{30}NO_9S$ $(M+H)^+$: Calc'd, 448.1636; Found, 448.1641. $C_{19}H_{29}NNaO_9S$ $(M+Na)^+$: Calc'd, 470.1455; Found, 470.1460.

*Synthesis of N-pivaloyl-a-D-5-thio-glucosamine (**12**) and N-pivaloyl-l,3,4,6-tetra-O-acetyl- α-D-5-thio-glucosamine (**12-OAc**)*—These compounds were prepared from **6** following general procedures A and B. For **12**: ^{1}H-NMR (500 MHz, D_2O): S 4.96 (d, *J* = 3.00 Hz, 1 H), 4.13 (dd, *J* = 10.5, 3.00 Hz, 1 H), 3.93 (dd, *J* = 12.0, 5.50 Hz, 1 H), 3.88 (dd, *J* = 12.0, 3.50 Hz, 1 H), 3.76 (t, *J* = 10.0 Hz, 1 H), 3.67 (t, *J* = 10.0 Hz, 1 H), 3.26 (ddd, *J*= 10.0, 5.50, 3.50 Hz, 1 H), 1.19 (s, 9 H). ^{13}C-NMR (125 MHz, D_2O): S 182.40, 74.05, 71.75, 71.34, 60.18, 58.16, 43.19, 38.57, 26.47. HRMS *(m/z)*: $C_{11}H_{22}NO_5S$ $(M+H)^+$: Calc'd, 280.1213; Found, 280.1203. $C_{11}H_{21}NNaO_5S$ $(M+Na)^+$: Calc'd, 302.1033; Found, 302.1025. For **12-OAc**: ^{1}H-NMR (500 MHz, $CDCl_3$): δ (ppm) 6.01 (d, *J*= 3.50 Hz, 1 H), 5.90 (d, *J* = 8.50 Hz, 1 H), 5.38 (dd, *J* = 10.5, 9.50 Hz, 1 H), 5.18 (dd, *J* = 10.5, 10.0 Hz, 1 H), 4.58-4.54 (m, 1 H), 4.34 (dd, *J* = 12.0, 5.00 Hz, 1 H), 4.03 (dd, *J* = 12.0, 3.00 Hz, 1 H), 3.48 (ddd, *J* = 11.0, 5.00, 3.00 Hz, 1 H), 2.17 (s, 3 H), 2.07 (s, 3 H), 2.04 (s, 3 H), 2.02 (s, 3 H), 1.09 (s, 9 H). ^{13}C-NMR (125 MHz, $CDCl_3$): δ (ppm) 177.87, 171.76, 170.54, 169.13, 168.59, 72.24, 71.59, 71.34, 61.14, 55.44, 39.81, 38.58, 27.19, 20.86, 20.62 (2 C), 20.50. HRMS *(m/z)*: $C_{19}H_{3o}NO_9S$ $(M+H)^+$: Calc'd, 448.1636; Found, 448.1636. $C_{19}H_{29}NNaO_9S$ $(M+Na)^+$: Calc'd, 470.1455; Found, 470.1453.

*Synthesis of N-hexonoyl-a-D-5-thio-glucosamine (**13**) and N-hexonoyl-l,3,4,6-tetra-O-acetyl- α-D-5-thio-glucosamine (**13-OAc**)*—These compounds were prepared from **6** following general procedures A and B. For **13**: ^{1}H-NMR (500 MHz, D_2O): δ (ppm) 4.96 (d, *J* = 2.50 Hz, 1 H), 4.10 (dd, *J* = 10.0, 3.00 Hz, 1 H), 3.93 (dd, *J* = 12.0, 5.50 Hz, 1 H), 3.88 (dd, *J* = 12.0, 3.00 Hz, 1 H), 3.70 (t, *J* = 9.50 Hz, 1 H), 3.66 (t, *J* = 9.50 Hz, 1 H), 3.27 (ddd, *J* = 9.50, 5.50, 3.50 Hz, 1 H), 2.28 (t, *J* = 7.50 Hz, 2 H), 1.63-1.56 (m, 2 H), 1.30-1.27 (m, 4 H), 0.86 (t, *J*= 7.00 Hz, 3 H). ^{13}C-NMR (125 MHz, D_2O): δ (ppm) 177.26, 74.05, 71.81, 71.42, 60.18, 58.02, 43.12, 35.66, 30.40, 25.04, 21.65, 13.20. HRMS *(m/z)*: $C_{12}H_{24}NO_5S$ $(M+H)^+$: Calc'd, 294.1370; Found, 294.1370. $C_{12}H_{22}NNaO_5S$ $(M+Na)^+$: Calc'd, 316.1189; Found, 316.1190. For **13-OAc**: ^{1}H-NMR (500 MHz, CDCl3): δ (ppm) 5.92 (d, *J* = 3.00 Hz, 1 H, H- 1), 5.72 (d, *J* = 8.50 Hz, 1 H, N-H), 5.38 (dd, *J* = 11.0, 10.0 Hz, 1 H), 5.18 (dd, *J* =11.0, 10.0 Hz, 1 H), 4.66-4.60 (m, 1 H), 4.35 (dd, *J* = 12.0, 5.00 Hz, 1 H), 4.01 (dd, *J* = 12.0, 3.00 Hz, 1 H), 3.46 (ddd, *J*= 11.0, 5.00, 3.00 Hz, 1 H), 2.17 (s, 3 H), 2.10-2.05 (m, 2 H), 2.06 (s, 3 H), 2.03 (s, 3 H), 2.02 (s, 3 H), 1.55-1.48 (m, 2 H), 1.31-1.25 (m, 2 H), 1.25-1.17 (m, 2 H), 0.85 (t, *J*= 7.50 Hz, 3 H). [I3]C-NMR (125 MHz, CDCl3): S 172.61, 171.62, 170.59, 169.13, 168.69, 72.65, 71.57, 71.32, 61.00, 54.90, 39.61, 36.46, 31.15, 25.20, 22.28, 21.08,

20.65, 20.51, 13.84. HRMS *(m/z)*: $C_{20}H_{32}NO_9S$ $(M+H)^+$: Calc'd, 462.1792; Found, 462.1791. $C_{20}H_{31}NNaO_9S$ $(M+Na)^+$: Calc'd, 484.1612; Found, 484.1612.

*Synthesis of N-(5-methylhexonoyl)-a-D-5-thio-glucosamine (**14**) and N-(5-methylhexonoyl)-l,3,4,6-tetra-O-acetyl-α-D-5-thio-glucosamine (**14-OAc**)*—These compounds were prepared from **6** following general procedures A and B. For **14**: ^{1}H-NMR (400 MHz, $CDCl_3$): δ (ppm) 5.95 (d, *J* = 2.80 Hz, 1 H), 5.68 (d, *J* = 8.80 Hz, 1 H), 5.38 (t, *J* = 10.4 Hz, 1 H), 5.18 (t, *J* = 10.4 Hz, 1 H), 4.67-4.61 (m, 1 H), 4.35 (dd, *J* = 12.0, 5.20 Hz, 1 H), 4.04 (dd, *J* = 12.0, 3.20 Hz, 1 H), 3.48 (ddd, *J* = 10.8, 4.80, 3.20 Hz, 1 H), 2.18 (s, 3 H), 2.10-2.00 (m, 2 H), 2.07 (s, 3 H), 2.04 (s, 3 H), 2.03 (s, 3 H), 1.56-1.48 (m, 3 H), 1.14-1.06 (m, 2 H), 0.86 (d, *J* = 6.80 Hz, 6 H). ^{13}C-NMR (100 MHz, $CDCl_3$): δ (ppm) 172.57, 171.65, 170.55, 169.12, 168.62, 72.72, 71.72, 71.50, 61.12, 55.07, 39.74, 38.26, 36.77, 27.78, 23.44, 22.41(2 C), 21.06, 20.67, 20.62, 20.50. HRMS (*m/z*): $C_{21}H_{34}NO_9S$ $(M+H)^+$: Calc'd, 476.1949; Found, 476.1942. $C_{21}H_{33}NNaO_9S$ $(M+Na)^+$: Calc'd, 498.1768; Found, 498.1764. For **14-OAc**: ^{1}H-NMR (600 MHz, MeOD): δ (ppm) 4.88 (d, *J* = 3.00 Hz, 1 H), 4.10 (dd, *J* = 10.2, 3.00 Hz, 1 H), 3.89 (dd, *J* = 11.4, 3.60 Hz, 1 H), 3.82 (dd, *J* = 11.4, 6.00 Hz, 1 H), 3.67 (dd, *J* = 10.2, 9.0 Hz, 1 H), 3.58 (dd, *J* = 10.2, 9.0 Hz, 1 H), 3.26 (ddd, *J* = 9.60, 6.00, 4.20 Hz, 1 H), 2.22 (d, *J* = 7.80 Hz, 2 H), 1.65-1.60 (m, 2 H), 1.59-1.53 (m, 1 H), 1.25-1.21 (m, 2 H), 0.89 (t, *J* = 6.60 Hz, 6 H). ^{I3}C-NMR (150 MHz, MeOD): δ (ppm) 176.36, 76.93, 73.60, 73.43, 62.68, 59.87, 44.90, 39.64, 37.34, 29.05, 24.92, 22.96. HRMS (*m/z*): $C_{13}H_{26}NO_5S$ $(M+H)^+$: Calc'd, 308.1526; Found, 308.1528. $C_{13}H_{25}NNaO_5S$ $(M+Na)^+$: Calc'd, 330.1346; Found, 330.1349.

*Synthesis of N-[(S)-methylvaleryl]-a-D-5-thio-glucosamine (**15**) and N-[(S)-methylvaleryl]-l,3,4,6-tetra-O-acetyl-α-D-5-thio-glucosamine (**15-OAc**)*—These compounds were prepared from **6** following general procedures A and B. For **15**: ^{1}H-NMR (400 MHz, $CDC1_3$): δ (ppm) 5.96 (d, 7= 3.20 Hz, 1 H), 5.71 (d, *J*- 8.40 Hz, 1 H), 5.38 (t, 7 = 10.8 Hz, 1 H), 5.19 (t, 7 = 10.8 Hz, 1 H), 4.67-4.61 (m, 1 H), 4.35 (dd, 7 = 12.0, 5.20 Hz, 1 H), 4.04 (dd, 7 = 12.0, 3.20 Hz, 1 H), 3.46 (ddd, 7= 10.8, 4.80, 3.20 Hz, 1 H), 2.17 (s, 3 H), 2.15-2.12 (m, 2 H), 2.07 (s, 3 H), 2.04 (s, 3 H), 2.03 (s, 3 H), 1.75-1.68 (m, 2 H), 1.33-1.23 (m, 1 H), 1.21-1.10 (m, 1 H), 0.88-0.83 (m, 6 H). ^{13}C-NMR (100 MHz, $CDCl_3$): δ (ppm) 172.08, 171.68, 170.54, 169.12, 168.62, 72.67, 71.59, 71.55, 61.11, 55.09, 43.86, 39.72, 32.10, 29.13, 21.02, 20.70, 20.61, 20.50, 18.92, 1 1.17. HRMS (m/z): $C_{12}H_{24}NO_5S$ $(M+H)^+$: Calc'd, 294.1370; Found, 294.1378. $C_{12}H_{23}NNaO_5S$ $(M+Na)^+$: Calc'd, 316.1189; Found, 316.1193. For **15-OAc**: ^{1}H-NMR (600 MHz, MeOD): δ (ppm) 4.88 (d, *J* = 2.40 Hz, 1 H), 4.11 (dd, *J* = 10.2, 3.00 Hz, 1 H), 3.89 (dd, *J* = 11.4, 3.60 Hz, 1 H), 3.83 (dd, *J* = 11.4, 6.00 Hz, 1 H), 3.68 (dd, *J* = 10.2, 9.0 Hz, 1 H), 3.57 (dd, *J* = 10.2, 9.0 Hz, 1 H), 3.27 (ddd, *J* = 9.60, 6.00, 4.20 Hz, 1 H), 2.24 (dd, *J* = 13.2, 6.0 Hz, 1 H), 2.04 (dd, *J* = 13.2, 7.8 Hz, 1 H), 1.89-1.83 (m, 1 H), 1.44-1.37 (m, 1 H), 1.26-1.19 (m, 1 H), 0.94 (d, 7= 6.60 Hz, 3 H), 0.92 (t, 7= 7.20 Hz, 3 H). ^{13}C-NMR (150 MHz, MeOD): δ (ppm) 175.83, 76.98, 73.62, 73.38, 62.68, 59.86, 44.90, 44.48, 33.75, 30.49, 19.54, 11.73. HRMS (*m/z*): $C_{20}H_{32}NO_9S$ $(M+H)^+$: Calc'd, 462.1792; Found, 462.1793. $C_{20}H_{31}NNaO_9S$ $(M+Na)^+$: Calc'd, 484.1612; Found, 484.1609.

*Synthesis of N-(2-cyclohexylacetyl)-a-D-5-thio-glucosamine (**16**) and N-(2-cyclohexylacetyl)-l,3,4,6-tetra-O-acetyl-α-D-5-thio-glucosamine (**16-OAc**)*—These compounds were prepared from **6** following general procedures A and B. For **16**: ^{1}H-NMR (600 MHz, $CDCl_3$): δ (ppm) 5.95 (d, *J* = 3.00 Hz, 1 H), 5.68 (d, *J* = 9.00 Hz, 1 H), 5.37 (t, *J* = 10.2 Hz, 1 H), 5.18 (t, *J* = 10.2 Hz, 1 H), 4.66- 4.60 (m, 1 H), 4.35 (dd, *J* = 12.0, 4.80 Hz, 1 H), 4.04 (dd, *J* = 12.0, 3.00 Hz, 1 H), 3.47 (ddd, *J* = 10.8, 4.80, 3.00 Hz, 1 H), 2.17 (s, 3 H), 2.06 (s, 3 H), 2.04 (s, 3 H), 2.02 (s, 3 H), 1.99 (dd, *J*= 13.8, 7.20 Hz, 1 H), 1.92 (dd, *J*= 13.8, 7.20 Hz, 1 H), 1.74-1.60 (m, 6 H), 1.28-1.20 (m, 2 H),

1.19-1.11 (m, 1 H), 0.88-0.81 (m, 2 H). ^{13}C-NMR (150 MHz, $CDCl_3$): δ (ppm) 171.81 , 171.66, 170.54, 169.12, 168.61, 72.66, 71.54, 71.50, 61.09, 55.06, 44.56, 39.68, 35.20, 32.85, 32.79, 26.02, 25.91, 21.02, 20.67, 20.61, 20.50. HRMS *(m/z)* $C_{14}H_{26}NO_5S$ $(M+H)^+$: Calc'd, 320.1526; Found, 320.1528. $C_{14}H_{25}NNaO_5S$ $(M+Na)^+$: Calc'd, 342.1346; Found, 342.1347. For **16-OAc**: ^{1}H-NMR (600 MHz, MeOD): S 4.88 (d, *J* = 2.40 Hz, 1 H), 4.10 (dd, *J* = 10.8, 3.00 Hz, 1 H), 3.89 (dd, *J* = 11.4, 3.60 Hz, 1 H), 3.82 (dd, *J* = 11.4, 6.00 Hz, 1 H), 3.67 (dd, *J* = 10.8, 9.0 Hz, 1 H), 3.57 (dd, *J*= 10.2, 9.0 Hz, 1 H), 3.26 (ddd, *J* = 10.2, 6.00, 4.20 Hz, 1 H), 2.11 (d, *J* = 7.20 Hz, 2 H), 1.80-1.68 (m, 5 H), 1.68-1.64 (m, 1 H), 1.32-1.25 (m, 2 H), 1.22-1.12 (m, 1 H), 1.03-0.96 (m, 2 H). ^{13}C-NMR (150 MHz, MeOD): δ (ppm) 175.57, 76.98, 73.65, 73.38, 62.69, 59.88, 45.11, 44.90, 36.89, 34.24, 27.42, 27.33, 27.32. $C_{22}H_{34}NO_9S$ $(M+H)^+$: Calc'd, 488.1949; Found, 488.1951. $C_{22}H_{33}NNaO_9S$ $(M+Na)^+$: Calc'd, 510.1768; Found, 510.1765.

*Synthesis of N-(1-naphthylacetyl)-a-D-5-thio-glucosamine (**17**) and N-(1-naphthylacetyl)-l,3,4,6-tetra-O-acetyl-α-D-5-thio-glucosamine (**17-OAc**)*—These compounds were prepared from **6** following general procedures A and B. For **17**: ^{1}H-NMR (400 MHz, acetone-D6 with some drops of MeOD): δ (ppm) 8.11 (d, *J* = 8.00 Hz, 1 H), 7.86 (d, *J* = 8.00 Hz, 1 H), 7.78 (d, *J* = 8.00 Hz, 1 H), 7.52-7.38 (m, 4 H), 4.87 (d, *J* = 2.40 Hz, 1 H), 4.12 (dd, *J* - 10.0, 2.40 Hz, 1 H), 4.05 (d, *J* = 2.80 Hz, 2 H), 3.86-3.74 (m, 2 H), 3.66 (t, *J* = 9.60 Hz, 1 H), 3.58 (t, *J* = 9.60 Hz, 1 H), 3.23-3.19 (m, 1 H). ^{13}C-NMR (100 MHz, acetone-D-6 with some drops of MeOD): δ (ppm) 173.45, 135.90, 134.40, 134.26, 130.35, 129.73, 129.37, 127.98, 127.53, 127.38, 126.12, 78.00, 74.28, 74.22, 63.63, 60.42, 45.45, 42.13. HRMS *(m/z)*: $C_{18}H_{22}NO_5S$ $(M+H)^+$: Calc'd, 364.1213; Found, 364.1220. $C_{18}H_{21}NNaO_5S$ $(M+Na)^+$: Calc'd, 386.1033; Found, 386.1035. For **17-OAc**: ^{1}H-NMR (400 MHz, $CDCl_3$): δ (ppm) 7.90-7.80 (m, 3 H), 7.56-7.51 (m, 2 H), 7.47 (t, *J*= 7.60 Hz, 1 H), 7.30 (d, *J* = 6.80 Hz, 1 H), 5.83 (d, *J* = 3.20 Hz, 1 H), 5.54 (d, *J* = 8.40 Hz, 1 H), 5.25 (t, *J* = 10.4 Hz, 1 H), 4.82 (t, *J* = 10.4 Hz, 1 H), 4.52-4.47 (m, 1 H), 4.26 (dd, *J* = 12.0, 4.80 Hz, 1 H), 4.00-3.90 (m, 3 H), 3.28 (ddd, *J* = 10.8, 4.80, 3.20 Hz, 1 H), 2.02 (s, 3 H), 1.94 (s, 3 H), 1.67 (s, 3 H), 1.66 (s, 3 H). ^{I3}C-NMR (100 MHz, $CDCl_3$): δ (ppm) 170.79, 170.45, 170.44, 169.03, 168.17, 133.92, 131.77, 130.25, 128.80, 128.69, 128.34, 127.11, 126.34, 125.66, 123.33, 71.95, 71.28, 70.97, 60.99, 55.30, 41.59, 39.57, 20.55, 20.37, 20.33, 20.03. HRMS (m/z): $C_{26}H_{30}NO_9S$ $(M+H)^+$: Calc'd, 532.1636; Found, 532.1633. $C_{26}H_{29}NNaO_9S$ $(M+Na)^+$: Calc'd, 554.1455; Found, 554.1450.

General procedures

All solutions containing salts and buffers were made from materials obtained from Bioshop Canada (Burlington, ON) and Sigma (St. Louis, MO) unless otherwise noted. The following antibodies were used for immunoblot analysis: anti-Sp1 (PEP2), and anti-β-actin (C4)), antibodies were obtained from Santa Cruz Biotechnology (Dallas, TX) and used at dilutions of 1:1000 and 1:3000, respectively. The anti-*O*-GlcNAc antibody (CTD110.6) was obtained from Covance (Princeton, NJ) and used at a dilution of 1:1500. The anti-C/EBP-α (EP708Y) and anti-C/EBP-β monoclonal antibodies (E299) were both purchased from Abcam (Cambridge, MA) and used at a dilution of 1:800. Goat-anti-mouse IgG/M and goat-anti-rabbit IgG/M fluorescently labeled secondary antibodies were obtained from Mandel Scientific (Guelph, ON); these secondary antibodies were typically used at dilutions of 1:10,000 to 1:20,000. An ultra-sensitive mouse leptin ELISA kit was obtained from Crystal Chem, Inc (Downers Grove, IL) and used exactly as recommended in the manufacturer's protocols. All leptin measurements were performed in duplicate. Recombinant mouse leptin was obtained from R & D Systems (Minneapolis, MN) in a lyophilized form. A stock solution of leptin at a concentration of 0.4 $mgmL^{-1}$ was prepared in filter-

sterilized (0.22 µm) 20 mM TRIS (base), pH 8.0 as recommended by the manufacturer; stock leptin solutions were stored at 4 ºC for no longer than three days. The purity of 5SGlcNHex (**12**) was confirmed to be higher than 95 percent by ^{1}H-NMR before any animal experiments were conducted. This inhibitor was diluted in sterile saline (0.9% NaCl; Hospira, Lake Forest, IL) to a final concentration of 30 mgmL^{-1}. This stock solution was filter-sterilized (0.22 µm) and stored either at 4 ºC (up to one week) or at -20 ºC (long-term). Protein concentrations were determined using the Bio-Rad (Hercules, CA) DC protein assay.

Capillary electrophoresis (CE) analysis of nucleotide sugars and sugar phosphates

Nucleotide sugars were extracted from 5SGlcNR-treated COS-7 cells and analyzed by CE precisely as previously described.[8] Sugar phosphates were analyzed by using an identical CE method in which the UV absorbance was monitored at 200 nm rather than 254 nm.

***In vitro* analysis of 5SGlcNR processing by GlcNAc-salvage enzymes**

The expression and purification of recombinant human GNK, AGM and AGX1 and 2 were performed as previously described. Recombinant human GlcNAc-6-phosphate deacetylase (NAGA) was cloned, expressed and purified as described by Bergfeld *et al*.[9] All enzymes were confirmed to be active on their natural substrates before conducting any experiments. NAGA digestions were performed in 50 mM TRIS, pH 7.5 containing 20 mM $MgCl_2$ and 2 mM GlcNAc-6-phosphate (Sigma). 5SGlcNHex-6-phosphate was prepared by exposing **12** (2 mM) to ATP (3 mM) in the presence of GNK at 37 ºC overnight; the resulting phosphorylated material was cleaned-up by solid phase extraction (SPE)[10] before testing with NAGA. After all, NAGA tests, the same SPE protocol was used to remove enzyme from the reaction mixture prior to CE analysis.

Cell culture

Chinese hamster ovary (CHO-K1) and African green monkey kidney (COS-7) cells were purchased from the ATCC (Manassas, VA) and mouse pre-adipocytes (3T3-L1) cells were a gift from Dr. Green at Harvard Medical School. COS7 and 3T3-L1 cells were grown in Dulbecco’s Modified Eagle’s Medium (DMEM) (Lonza; Basel, SW) supplemented with 10% fetal bovine serum (FBS) (Gibco/Life Technologies; Carlsbad, CA). CHO-K1 cells were grown in DMEM: Ham’s F12, 1:1 (v/v) media (Gibco) containing 5 % FBS. Cells were passed twice weekly using trypsin/EDTA (Gibco) as previously described. Cells were incubated in a humidified atmosphere containing 5% CO_2 at 37 ºC. To different 3T3-L1 cells into mature adipocytes, preadipocytes at a confluency of 70% were switched to DMEM containing 10 % bovine serum (BS; Gibco) for 24 h. The cells were then cultured in DMEM containing 10% FBS, 120 µgmL^{-1} isobutylmethylxanthine (IBMX; Sigma), 0.37 µgmL^{-1} dexamethazone (DEX; Sigma), and 1 µgmL^{-1} Humulin insulin (Lilly; Indianapolis, IN). The medium was carefully replaced every two days for one month until the majority of cells were fully differentiated.[7,11]

Inhibitors were tested in cells that were at 70-80 % confluence unless noted. In all cases, fresh culture media were added prior to the addition of inhibitors. Peracetylated compounds were added to cells from stock solutions in DMSO and care was taken to ensure that the final DMSO concentration did not exceed 0.1% (v/v). Stock solutions of deprotected inhibitors (such as **12**) were prepared in cell culture medium and passed through a syringe filter (0.22 µm) before use. All stocks were stored long-term at -20 ºC. To determine which analogues most potently decrease *O*-GlcNAc levels, all analogues were initially screened in CHO K1 cells at a final concentration of 50 µM for 24 h. Likewise, time-response experiments were performed at inhibitor concentrations of 50 µM unless indicated. Cells extracts were prepared for immunoblot analysis by removing the

media and washing the cells with 1 x 1 mL cold phosphate-buffered saline (PBS; Lonza). Cells were then incubated on ice for 15 min in 1x radioimmunoprecipitation (RIPA) buffer [50 mM TRIS (H^+), pH 7.5, 150 mM NaCl, 0.1% SDS, 0.5% sodium deoxycholate and 1% NP-40] containing 1x protease inhibitor cocktail (Roche; Basel, SW), scraped of plates and sonicated (10 s, 20% duty, 4 ^oC) using a Sonic Dismembrator, Model 500 (Fisher Scientific International, Inc; Hampton, NH). Cell lysates were clarified by centrifugation (10,000 x g, 10 min, 4 ^oC); supernatants were stored at -20 ^oC until analysis.

To analyze the effects of $Ac_4$5SGlcNAc (**3**-OAc) and 5SGlcNHex (**12**) on glucose (Glc)- or GlcNAc-stimulate leptin secretion in fully differentiated 3T3-L1 adipocytes, cells were starved overnight in Glc-free media before they were switched to media containing 5 mM GlcNAc with or without **3**-OAc (200 μM) and **12** (100 μM). An identical experiment was performed by using 5 mM Glc to stimulate leptin secretion. In both cases, control samples received sugar-free media. Cells were grown in the new media of these additional time points to allow for the accumulation of sugar-stimulated leptin in the culture media. After the various time points, the media were collected, and cell lysates were prepared as described above. The media were supplemented with 1x protease inhibitor cocktail (Roche) and conditioned by centrifugation (200 x g, 10 min, 4 ^oC) to remove dead cells. Media were further concentrated using a 0.5 mL Amicon Ultra centrifugal concentrator, NMWCO = 3 kDa (Millipore; Billerica, MA) prior to the analysis of leptin levels using a mouse leptin ELISA kit (Crystal Chem).

To test the 5SGlcNAc does not inhibit glucose uptake and cell viability in 3T3-L1 adipocytes, DMSO and peracetylated 5SGlcNAc (**3**-OAc) or 5SGlucose (**5SGlc**-OAc, 200 μM) treatment cells (8, 16, 24, 48, and 72 h) were seeded on black, micro clear 96-well plates at a concentration of ~25,000 cells/well, were incubated at 37 degree for 10 min with PBS containing BSA 1 $mgmL^{-1}$ and 150 μgmL^{-1} of the fluorescent glucose analog 2-NBDG (Thermo Fisher Scientific) in the no-Glc and Na-pyruvate DMEM medium. Control cultures were treated with 4 μgmL^{-1} insulin. After incubation, cells were washed thrice with PBS, and the resultant fluorescence was measured (Excitation at 485 nm and Emission at 535 nm) using a fluorescent microplate reader.

Animal care

All experiments conducted with animals were approved by the Simon Fraser University University Animal Care Committee (UACC) (protocol number 981C-10) in accordance with the guidelines from the Canadian Council on Animal Care (CCAC). Female C57BL/6 five-week-old mice were obtained from Charles River Laboratories International, Inc. (Wilmington, MA) and allowed to acclimatize for one week before any experiments. Rooms were kept at 22 ^oC and maintained at a constant 12 h light (beginning at 7:00 AM) and 12 h dark cycle. The mice were housed in separate cages. Care was taken to ensure that mice had plenty of bedding and nesting material since it is known that mice deficient in leptin production or signaling display impaired thermoregulation.[12] The mice were allowed access to food and drink *ad libitum*. In all studies, mice were subjected to intraperitoneal (IP) injections.

Several pilot studies in mice were conducted. First, mice (n = 2) were treated with **12** at 300 $mgkg^{-1}$ and subsequently sacrificed at 0, 0.5, 2, 5, 8, 12, 16, 24, and 48 h post-injection. Likewise, in a dose-response study, mice (n = 2) were injected with **12** at a concentration of 0, 3, 10, 30, 100, and 300 $mgkg^{-1}$ for 16 h. Finally, to assess the tissue-specific effects of **12**, mice (n = 3) were dosed once for 16 h at 300 $mgkg^{-1}$ prior to sacrifice. Mice in these pilot studies exhibited signs of apparent hyperphagia, that is, their stomachs were full of rodent chow, and they had deficient plasma leptin levels. To further assess the effects of **12** on nutrient sensing and the

regulation of feeding behavior *via* the leptin pathway a follow-up experiment was performed in which mice were injected with vehicle (saline) or **12** (300 $mgkg^{-1}day^{-1}$) for two days. All mice were injected at the same time every day (13:00) and closely monitored for their reactions. Mice (n = 3) from each group were sacrificed 24 or 48 h after receiving their final dose of inhibitor and tissues were collected as described. For low dose **12** used, the effect of treatment of mice (n = 3) with the compound on *O*-GlcNAc levels in various tissues and leptin levels in plasma will be evaluated 0, 0.5, 2, 5, 8, 12, 16, 24, and 48 hours after the first dosing with 50 $mgkg^{-1}$ of the compound and 5, 12, 24 for vehicle (saline) alone.

Preparation of mouse tissues for immunoblot analysis

Following the completion of experiments, mice were sacrificed in a carbon dioxide chamber. Blood was immediately drawn by cardiac puncture and transferred to chilled, pre-heparinized tubes. Plasma was collected from the blood by centrifugation (10 min, 4 ^{o}C, 1300 x g). Mice were carefully dissected, and the brain, lungs, heart, pancreas, liver, kidneys, muscle, stomach and visceral fat were collected. All tissues and the sera were immediately flash frozen in liquid nitrogen; samples were kept at -80 ^{o}C freezer for long-term storage.

Tissue supernatants were prepared for immunoblot analysis by thawing them on the ice and removing a portion with a clean scalpel. This piece of tissue was snap frozen with liquid nitrogen, and ground into a powder using a mortar and pestle which had similarly been pre-chilled. The powered tissue was suspended in 600 µL of 1 x radioimmunoprecipitation assay (RIPA) buffer containing a 1 x protease inhibitor cocktail (Roche) and minced for 20 s using a T-18 Ultra-Turrax tissue homogenizer (IKA; Wilmington, NC). The solution was further sonicated using a Sonic Dismembrator, Model 500 (Fisher) at 20% duty for 15 s. The tissue solutions were left at 4 ^{o}C for 30 min to completely solubilize all proteins before they were clarified by centrifugation (20 min, 4 ^{o}C, 15,000 x g). The protein concentrations of the supernatants measured, and the volumes of all samples were more RIPA and Laemmli buffer to make final solutions 2 $mgmL^{-1}$. Samples in Laemmli buffer were boiled for 10 min and stored at -20 ^{o}C; any remaining supernatants were stored at -80 ^{o}C.

Immunoblotting

Cell lysates and tissue samples (40 - 50 µg/lane) were resolved by SDS polyacrylamide gel electrophoresis (SDS-PAGE) on 10% acrylamide gels and electrophoretically transferred to nitrocellulose membranes (Bio-Rad). The membranes were blocked for 1 hour in 5% BSA (Bioshop) in PBS at room temperature (RT). The membranes were blotted overnight at 4 ^{o}C with the appropriate primary antibodies in PBS containing 0.1 % Tween-20 (PBS-T) containing 5 % BSA. Membranes were extensively washed over 1 h with three changes of PBS-T, re-blocked, and incubated with appropriate horseradish peroxidase-labeled secondary antibodies for 1 h at RT. The membranes were washed for an additional hour with PBS-T and developed using the Pierce™ ECL Western blotting substrate (ThermoFisher) while the transcription factors (Sp1 and C/EBPs) were scanned using a Licor® Odyssey infrared imaging system (Mandel) after probing with the appropriate fluorescently-labeled secondary antibodies. Actin levels were determined by re-blotting the membranes after they had been stripped by incubation in 0.5 M TRIS (H^+), pH 6.8, containing 1% SDS and 1% β-mercaptoethanol (Bioshop) for 15 min at room temperature. The fluorescent signals for *O*-GlcNAc and Sp1 were normalized to the relative actin levels obtained from the re-probed membranes. To account for the fact that *O*-GlcNAc levels vary significantly across different tissues, and to permit the determination of the relative effects of **12** in various tissues, the untreated controls for each tissue type were adjusted to 100%. Lectin blotting was

accomplished using the same procedure with the following modifications: only 5 μg protein was loaded per lane, and BSA blocking was avoided until the addition of secondary staining reagents, namely fluorescently-labeled streptavidin (Mandel). Lectin blots were scanned using a Licor® Odyssey infrared imaging system.

Statistical analysis

All replicate data are reported as averages ± the standard error of the mean (SEM). Statistical analyses were performed using Microsoft Excel. Differences between treated and untreated animals were assessed using the Student's t-test (paired, 2-tailed); differences were considered significant if $p < 0.05$.

SUPPLEMENTAL REFERENCES

[1] J. Wang, R. Liu, M. Hawkins, N. Barzilai, L. Rossetti, *Nature* **1998**, *393*, 684–688.
[2] W. M. Mueller, F. M. Gregoire, K. L. Stanhope, C. V. Mobbs, T. M. Mizuno, C. H. Warden, J. S. Stern, P. J. Havel, *Endocrinology* **1998**, *139*, 551–558.
[3] P. Zhang, E. S. Klenk, M. A. Lazzaro, L. B. Williams, R. V. Considine, *Endocrinology* **2002**, *143*, 99–106.
[4] H. J. Goldberg, C. I. Whiteside, G. W. Hart, I. G. Fantus, *Endocrinology* **2006**, *147*, 222–231.
[5] I. Han, J. E. Kudlow, *Mol. Cell. Biol.* **1997**, *17*, 2550–2558.
[6] M. M. Mason, Y. He, H. Chen, M. J. Quon, M. Reitman, *Endocrinology* **1998**, *139*, 1013–1022.
[7] K. Ishihara, I. Takahashi, Y. Tsuchiya, M. Hasegawa, K. Kamemura, *Biochem. Biophys. Res. Commun.* **2010**, *398*, 489–494.
[8] T. M. Gloster, W. F. Zandberg, J. E. Heinonen, D. L. Shen, L. Deng, D. J. Vocadlo, *Nat. Chem. Biol.* **2011**, *7*, 174–181.
[9] A. K. Bergfeld, O. M. T. Pearce, S. L. Diaz, T. Pham, A. Varki, *J. Biol. Chem.* **2012**, *287*, 28865–28881.
[10] W. F. Zandberg, N. Gao, J. Kumarasamy, M. A. Lehrman, N. G. Seidah, B. M. Pinto, *ChemBiochem* **2012**, *13*, 392–401.
[11] B. C. Reed, M. D. Lane, *Proc. Natl. Acad. Sci. U. S. A.* **1980**, *77*, 285–289.
[12] M. A. Pelleymounter, M. J. Cullen, M. B. Baker, R. Hecht, D. Winters, T. Boone, F. Collins, *Science* **1995**, *269*, 540–3.
